# Development and validation of a machine learning-driven mitochondrial gene signature for the diagnosis of breast cancer

**DOI:** 10.3389/fimmu.2025.1712089

**Published:** 2025-12-03

**Authors:** Siyu Tong, Fei Teng, Weijia Kong, Xuanhe Tian, Dong Guo, Meng Liu, Jian Ren

**Affiliations:** 1 College of Traditional Chinese Medicine, Shandong University of Traditional Chinese Medicine, Jinan, China; 2 Beijing University of Chinese Medicine Third Affiliated Hospital, Beijing, China; 3 Guang’anmen Hospital, China Academy of Chinese Medical Sciences, Beijing, China; 4 Graduate School, Beijing University of Chinese Medicine, Beijing, China; 5 First School of Clinical Medicine, Shandong University of Traditional Chinese Medicine, Jinan, China; 6 Shandong University of Traditional Chinese Medicine, Jinan, China; 7 Oncology Department of Integrated Traditional Chinese and Western Medicine, China-Japan Friendship Hospital, Beijing, China

**Keywords:** breast cancer, mitochondrial gene, biomarker, machine learning, immunohistochemistry

## Abstract

**Background:**

Breast cancer (BC) ranks among the most prevalent malignant tumors in women globally, with mitochondrial dysfunction constituting one of its pathogenic mechanisms.

**Objectives:**

To investigate the relationship between mitochondrial function-related genes and BC progression.

**Methods:**

We identified BC differentially expressed genes via the GEO database, constructed a weighted co-expression network to determine BC pathogenesis-related key modules. Using 113 machine learning algorithms and MitoCarta mitochondrial genetics data, we developed a mitochondrial gene-based diagnostic model. GO/KEGG enrichment analyses delineated BC-related biological processes of mitochondrial genes, offering clues for understanding BC mechanism. High-throughput tissue chip and Immunohistochemistry (IHC) validated key genes’ local expression in tissues. CiberSort immune infiltration analysis highlighted NK and T cells’ role in BC; single-cell analysis identified gene expression patterns across tumor microenvironment cell types. Computational drug prediction and molecular docking explored targeted therapeutic candidates. Additionally, we conducted molecular dynamics simulations.

**Results:**

The glmBoost+LDA model had the highest C-index (0.947) in the validated cohort, including 18 potential BC biomarkers (e.g., ACADS, AUC = 0.810; AIFM2, AUC = 0.806). The results of experimental validation showed that the expression score of ACADS in cancerous tissues was significantly lower than that in adjacent non-cancerous tissues. Immune infiltration and single-cell analyses emphasized the crucial roles of NK cells and T cells in BC. Disulfiram and eugenol were predicted as potential therapeutics and validated by docking. Molecular dynamics simulations validated that Eugenol exhibits strong binding interactions with the target proteins AIFM2 and ACADS.

**Conclusions:**

This study identifies mitochondrial gene signatures associated with BC and proposes a computational model distinguishing tumor from normal tissue. These findings offer potential leads for future biomarker development but require additional clinical and functional validation.

## 1 Introduction

Breast cancer (BC) ranks among the most prevalent malignant tumors worldwide (1). In China, it is the most frequently diagnosed malignancy among women, constituting the leading cause of cancer mortality in females aged 15–44, with mortality rates continuing to rise (2, 3). Current BC treatments lack effective targets and face challenges of drug resistance and toxicity (4). Although combination therapy integrating targeted and immunotherapy shows great promise, predictive biomarkers for treatment efficacy remain scarce, making it difficult to forecast patient outcomes (5).

Mitochondria are vital organelles within cells, primarily responsible for numerous physiological processes including cellular energy metabolism and apoptosis regulation (6). In recent years, mitochondrial research has emerged as a significant direction within the life sciences, with notable advances particularly in tumor energy metabolism (7) and the regulation of immune cells (8). Mitochondrial dysfunction is closely associated with the initiation, progression, and treatment of BC. In BC cells, inhibition of mitochondrial oxidative metabolism induces apoptosis (9). Mitochondrial dysfunction in cancer produces lactic acid and promotes the growth of BC tumors (10). Moreover, the process of mitochondrial autophagy may play a pivotal role in BC treatment (11). Research by C C Cook et al. has revealed that mutations, deletions, and depletion of the mitochondrial genome can determine the advanced-stage phenotype of BC (12).

Meanwhile, machine learning methods have demonstrated strong data analysis capabilities in the field of oncology research. By integrating multiple algorithms, they can efficiently identify potential biomarkers from large-scale datasets. Currently, such methods have been widely applied to the mechanism research of various diseases, and play a crucial role especially in biomarker discovery and diagnostic model construction (13). In pancreatic cancer research, Yunju Jo et al. constructed a machine learning model based on multiple transcriptome datasets, successfully screening out the secretory protein-coding gene SCG5. The expression level of SCG5 is significantly reduced in pancreatic cancer tissues and is negatively correlated with tumor progression (14). In the field of colorectal cancer, machine learning methods have been successfully applied to gene expression analysis (15), prognostic prediction (16), and therapeutic target identification (17). However, current mitochondrial research in BC remains largely confined to conventional experimental methods, including *in vitro* cellular studies (18) and animal model investigations (19), with a lack of research on analyzing mitochondrial function-related genes and BC using machine learning. Compared with traditional statistical methods, machine learning can handle complex nonlinear relationships and large-scale data. By integrating multiple machine learning algorithms, a multivariate prediction framework can be constructed to screen out the optimal model for identifying potential biomarkers. This enables us to more comprehensively analyze the relationship between mitochondrial function-related genes and BC progression.

This study aims to develop a computational model capable of distinguishing between tumor and normal tissue for BC diagnosis. This is achieved by integrating multiple machine learning approaches based on mitochondrial-related gene biomarkers. Key gene biomarkers were experimentally validated. By analyzing key genes and their immune-regulatory mechanisms, we seek to explore novel directions for personalized targeted therapies against BC in clinical practice, thereby providing fresh perspectives and tools for the diagnosis and treatment of BC.

## 2 Materials and methods

### 2.1 Data sources for genes associated with mitochondrial function in BC

This study collected BC datasets from the GEO database (https://www.ncbi.nlm.nih.gov/gds/). Six specific datasets were identified: GSE10810, GSE33447, GSE7904, GSE42568, GSE45581, and GSE103512. These have been confirmed to originate from four distinct microarray platforms (corresponding GPL numbers are listed in **Supplementary Table S1**). During the preprocessing workflow, to ensure data compatibility across different platforms, probe IDs were first mapped to HGNC-approved standard gene symbols based on the official GPL annotation files corresponding to each dataset. Entries that could not be mapped or exhibited one-to-many/many-to-many ambiguities were removed. For multiple probes corresponding to the same gene, the limma package’s “avereps” function is employed to calculate the mean probe signal at the gene level, thereby generating a “gene × sample” expression matrix for each sample. Where multiple probes mapped to the same gene, the median of these values was designated as the gene’s expression level. This process formed a refined expression matrix by converting probe identifiers to their respective gene symbols. The expression matrix was further standardized using robust multi-array averaging (RMA). For datasets with missing values, multiple imputation was performed using k-nearest neighbors (KNN) weighted averaging.

To stabilize variance and reduce intensity-dependent bias, we automatically determine whether log transformation is required based on quantile thresholds: when the 99th quantile of expression values exceeds 100, or when (maximum-minimum range >50 and the 25th quantile >0), the expression matrix undergoes log2(x+1) transformation; Subsequently, limma’s “normaliseBetweenArrays” function is applied to perform quantile normalization, ensuring consistent intensity distributions across samples. Within each dataset, samples are annotated based on predefined grouping information (Control vs. Treat), and the corresponding normalized matrix is output (“GSE××××××.normalise.txt”).

**Figure 1:**
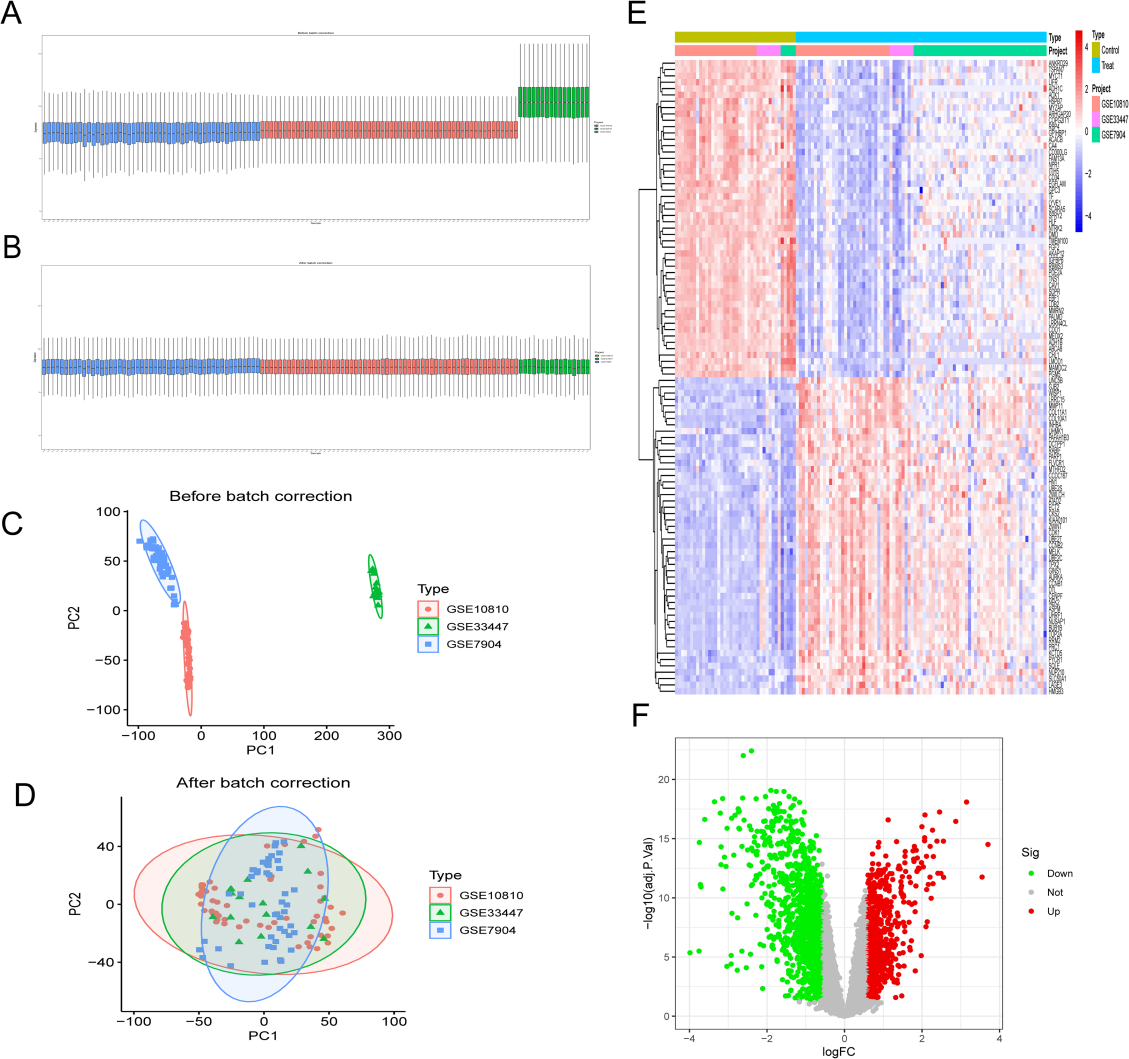
Identification of DEGs in BC.**(A)** Box plot prior to batch-debalancing; **(B)** Box plot after batch-debalancing; **(C)** PCA plot prior to batch-debalancing; **(D)** PCA plot after batch-debalancing; **(E)** Volcano plot of DEGs after batch-debalancing; **(F)** Heatmap of DEGs after batch-debalancing.

The expression profiles from the three pre-processed datasets—GSE10810, GSE33447, and GSE7904—were merged to form the training set. Subsequently, the “comBat” function within the sva package was employed to eliminate batch effects between datasets. To assess batch effects, we conducted principal component analysis (PCA). The three datasets GSE42568, GSE45581 and GSE103512 were standardized according to their respective platform characteristics and employed as independent validation cohorts for the model. **Figure 1** presents a visual representation of our study. Human mitochondrial gene sets were downloaded from the MitoCarta database (https://personal.broadinstitute.org/scalvo/MitoCarta3.0/human.mitocarta3.0.html), yielding 1,136 mitochondrial-associated immune-related genes after removing duplicates.

### 2.2 Analysis of differentially expressed genes

Following standardization and normalization of the dataset, we employed the R package “Limma” to identify DEGs from the merged training dataset. The screening criteria were: p.adjust value <0.05 and |log2FC| > 1.5. We employed the FDR method to adjust the p.adjust values, which served as the screening criteria. To visually illustrate the expression patterns of DEGs, we generated volcano plots and heatmaps using the “ggplot2” and “pheapmap” packages in R software respectively.

### 2.3 WGCNA analysis

WGCNA is employed to identify highly correlated gene modules, summarize the interconnections between modules and their associations with external sample characteristics, and identify candidate biomarkers or therapeutic targets. We constructed co-expression networks using the R package “WGCNA”. The co-expression network was built using genes retained from the merged dataset where the standard deviation exceeded 0.5, with a minimum module size of 60 genes. Specifically, we first preprocessed the sample data and removed outliers. Subsequently, we employed the “WGCNA” software package to construct a correlation matrix. We selected the optimal soft threshold to convert the correlation matrix into an adjacency matrix, from which we generated a topological overlap matrix (TOM). Utilizing phase difference metrics based on TOM, we classified genes with similar expression patterns into gene modules via average linkage hierarchical clustering (20). Finally, correlations between each module and clinical characteristics were assessed. Gene significance and module membership were calculated to measure the relevance and significance of genes within biological modules and clinical information. Key modules and genes were extracted for subsequent analysis.

### 2.4 Enrichment analysis of BC DEGs

To precisely identify hub genes, we first intersected the 1,256 DEGs obtained with the 3,395 WGCNA key module genes, thereby determining 599 differentially expressed key BC genes. Subsequently, we performed an intersection analysis between this key BC gene set and 1,136 mitochondrial function-related genes, screening out the final hub genes for further investigation. Subsequently, using the “Hs.eg.db” and “clusterProfiler” packages within R software, gene ontology (GO) functional and Kyoto encyclopedia of genes and genomes (KEGG) enrichment analyses were performed on the hub genes. This yielded multiple signaling pathways with P < 0.05, revealing biological processes (BP), cellular component (CC), molecular function (MF), and pathways implicated by DEGs. Visualization of results was achieved using the “ComplexHeatmap”, “RColorBrewer”, “ggplot2”, and “enrichplot” packages.

### 2.5 Exploration of 12 machine learning algorithms for constructing a mitochondrial gene-related BC diagnostic model

This study integrated twelve machine learning algorithms, including Stepwise Logistic Regression (StepGLM), Random Forests (RF), Gradient Boosting (GBM), the Least Absolute Shrinkage and Selection Operator (Lasso), Generalized Linear Model Boost (GlmBoost), Naive Bayes (NaveBayes), Support Vector Machines (SVM), Elastic Net (Enet), Linear Discriminant Analysis (LDA), Partial Least Squares Regression Generalized Linear Model (PlsRglm), Ridge Regression, and Extreme Gradient Boosting (XGBoost) (21). The performance of twelve machine learning algorithms in diagnostic modelling for bioinformatics data was evaluated using the R package system. Prior to modelling, batch effects were corrected for the GSE10810, GSE33447, and GSE7904 datasets using the “ComBat” function within the “sva” package. Input features defined as the intersection of “DEGs” (“limma” package, |Log2FC| > 1.5, p.adj<0.05) with WGCNA key module genes (MEturquoise module, r=-0.4, P = 5e-6) and mitochondrial genes (MitoCarta 3.0).

Based on the aforementioned feature sets, 113 algorithmic combinations were explored through the paired integration of 12 distinct foundational algorithms. We constructed all possible pairwise combinations, yielding a total of 113 model configurations by employing a segmentation strategy based on the α-value within the first algorithm.

We have constructed a unified binary classification machine learning framework. Data from each cohort may optionally undergo centering and standardization (scaleData, centreFlags/scaleFlags, default FALSE). The implemented algorithms include: Penalized logistic regression (Glmnet, selecting optimal λ via 10-fold cross-validation; Lasso: α=1, Ridge: α=0, Enet using custom α); StepGLM (direction configurable as forward, backward, or both); SVM (probability=TRUE); lDA; GlmBoost (retraining after determining optimal iteration steps via cvrisk); PlsRglm (model=“pls-glm-logistic”, sparse=TRUE); RF (n-tree=1000, importance=TRUE); GBM (distribution=“bernoulli”, optimal number of trees determined via cross-validation); XGBoost (5-fold cross-validation, objective=“binary:logistic”, retrained based on optimal number of rounds); NaveBayes.

Feature selection is uniformly implemented via the ExtractVar function: Glmnet, Glmboost, and PlsRglm remove variables with zero coefficients; stepwise regression returns variables entering the final model; random forest and GBM retain variables with relative importance > 0; SVM, LDA, XGBoost, and NaveBayes utilize all features. All models record subFeature for unified prediction input. “RunML” supports both mode=“Model” (outputting final models) and mode=“Variable” (outputting feature sets), facilitating sequential workflows where screening precedes modelling. During prediction, “CalPredictScore” calculates probability or risk scores (converted to binary outcomes by “PredictClass” using a 0.5 threshold). “RunEval” computes AUC for each cohort, with optional training set pooling for evaluation. Result visualization is achieved via “SimpleHeatmap” or “ComplexHeatmap”.

For ensemble methods such as “Lasso+SVM” and “GlmBoost+Ridge”, the former is first employed to select features, followed by modelling with the latter. By leveraging the unique capabilities of these foundational algorithms, we integrated 113 diagnostic models, significantly enhancing predictive efficacy in breast cancer diagnosis. The optimal model was determined on the training set. Finally, independent validation was conducted using external datasets GSE42568, GSE45581, and GSE103512. The genetic information included in all models is presented in **Supplementary Table S2**.

During model training and evaluation, the receiver operating characteristic (ROC) curve was employed to assess model performance, with the area under the curve (AUC) serving as an indicator of classification capability. Throughout the analysis, various algorithm combinations were thoroughly compared, and the model yielding the highest mean AUC was selected as the optimal model for BC mitochondrial-associated gene diagnostic feature selection and classification prediction.

Model optimization employed 10-fold cross-validation to determine the iteration count yielding the minimum validation error. The final model was trained using this optimal tree configuration to ensure robust generalization performance (22).

### 2.6 Assessing the diagnostic value of key biomarkers for BC

Employ the “pROC” package within R software to plot ROC curves, thereby evaluating the diagnostic value of identified biomarkers. Further validate the expression levels and diagnostic efficacy of candidate markers within the validation datasets GSE42568, GSE45581, and GSE103512. The study employed confusion matrices to evaluate predictive model performance. Genes identified within key modules were visualized using volcano plots generated via the “ggplot” package. Box plots illustrated gene expression differences between experimental and control groups within these key modules. Individual ROC curves were plotted for each gene within the key modules to assess their diagnostic value. Additionally, to validate the robustness and generalizability of the model, we performed cross-platform validation using RNA-seq data from the TCGA-BRCA cohort.

### 2.7 GSEA enrichment analysis

GSEA performs gene functional enrichment analysis on gene sets between two groups. GSEA identifies a set of genes sharing common biological functions or regulatory characteristics, exhibiting coordinated expression changes within the dataset even when these alterations are subtle. This approach enables the inclusion and analysis of genes that may display only minor expression differences yet share significant biological implications. The R package “clusterprofiler” was employed to perform GSEA on DEGs. The threshold was set at p < 0.05, with the reference gene set “c2.cp.kegg.Hs.symbols.gmt” selected.

### 2.8 High-throughput tissue chip and immunohistochemistry analysis

Utilizing the Human Protein Atlas (HPA) database (http://www.proteinatlas.org/) and immunohistochemical techniques, further evaluate the local expression patterns of key genes within tissue samples. We further verified the expression of ACADS in BC and its association with clinical and pathological features using high-throughput tissue chips and immunohistochemistry technology. The high-throughput tissue chips contained 60 cases of BC and 60 adjacent tissues (lot number: HBreD120CS01, Shanghai Xinsu Biotechnology Co., Ltd., Shanghai, China). After initial inclusion, 4 pairs of non-cancerous tissue samples were excluded based on histopathological diagnosis. All samples were taken from female patients, including 40 cases of invasive ductal carcinoma (IDC) and 16 cases of non-specific invasive carcinoma (NIDC). All tissue samples were collected prior to any treatment, and informed consent was obtained before collection.

All tissue samples were embedded in paraffin. The slides were placed in a constant temperature drying oven at 63°C and baked for one hour. Dewaxing was completed using a fully automatic dyeing machine. The slides were placed into the antigen retrieval apparatus, the appropriate program was selected, and antigen retrieval was initiated. After antigen retrieval, the slides were immersed in distilled water at room temperature and allowed to cool naturally for more than 10 minutes. The slides were incubated overnight at 4°Cwith ACADS polyclonal antibody (Cat No. 16623-1-AP, 1:1000). The slides were processed using the DAKO Autostainer Link 48 with EnVision FLEX+ Mouse, High pH reagent (K8002). Hematoxylin staining was performed for approximately 1 minute, followed by rinsing with tap water for 5 minutes. The slides were dried at room temperature and covered with neutral resin. The primary antibody was replaced by PBS as a negative control. The slides were scanned using the Aperio Scanner (Aperio XT, LEICA). Two pathologists, blinded to clinical information and each other’s assessments, conducted a double-blind evaluation of the staining results based on the proportion of positive staining in the sections.

### 2.9 Evaluation of immune cell subpopulation abundance and expression differences

Analyze the abundance and correlation of immune infiltrating cells using the CIBERSORT deconvolution algorithm. The Cibersort method primarily relies on the LM22 immune cell subtype expression matrix. Qualitative and quantitative analyses of this matrix were performed using the “CIBERSORT” R package. Immune infiltration results were filtered and retained at P<0.05. Box plots illustrating immune infiltration-related differential genes were plotted. Subsequently, Spearman correlation analysis was employed to examine relationships between immune cells and hub genes.

### 2.10 Single-cell analysis

The specific dataset GSE161529 was sourced from the GEO database. Data preprocessing initially involved cell-level filtering to exclude low-quality cells exhibiting mitochondrial gene expression exceeding 15%, total UMI counts below 500, or fewer than 250 detected genes; environmental RNA contamination and technical batch effects were subsequently corrected. Subsequently, Principal Component Analysis (PCA) was employed to resolve cell type and state similarities. The PCA scatterplot revealed the distribution characteristics of tumor samples within the principal component space. Further application of UMAP (Uniform Manifold Approximation and Projection) enabled clustering and separation of cell subpopulations, with the resulting endpoint plot illustrating the diversity of cell types or potential subtypes. The cell-cell interaction network diagram is used to explore the interaction frequency between T cells with different ACADS gene expression profiles and various cell types, highlighting the key cellular pathways involved in signal transduction.

### 2.11 Prediction of potential drugs for hub genes and molecular docking

Predict candidate therapeutic drugs for BC using the DGIdb database (https://www.dgidb.org/). Implement gene-to-drug enrichment analysis via the “clusterProfiler” and “org.Hs.eg.db” packages, subsequently visualizing enrichment results using “enrichplot” and “ggplot2”.

Perform molecular docking between predicted drugs and pivotal gene biomarker. Download the three-dimensional co-crystal structures of the target proteins GSR, PMAIP1, ACADS, and AIFM2 from the PDB database (https://www1.rcsb.org/), with PDB IDs 1DNC (23), 3MQP (24), 7T0A (25), and 8YOQ (26) respectively. Preprocess the downloaded structures using PyMOL 2.3.0 software, performing steps such as dehydration and hydrogenation. Subsequently, molecular structure files for the ligands Disulfiram and Eugenol were downloaded from the PubChem database. Molecular mechanics optimization was performed on the conformations of these small molecules using Chem3D (version 2020) software, ultimately yielding the energetically minimized optimal conformations. Using AutoDock Tools 1.5.7, the pre-processed target proteins were further processed to generate pdbqt files. Pocket analysis was conducted on the co-crystal structures of the four selected proteins. Ultimately, the native substrate from their PDB structures was selected as the docking pocket center. AutoDock Vina v.1.2.0 software was employed to perform molecular simulation docking between the target proteins and ligand molecules. The docking algorithm was set to the Lamarckian genetic algorithm, with semi-flexible docking mode, exhaustiveness set to 16, and the maximum number of conformations output set to 9.

### 2.12 Molecular dynamics simulation of protein-ligand complexes

This study employed GROMACS 2022 for molecular dynamics simulations, with force field parameters generated via GROMACS’ pdb2gmx utility. The GAFF2 force field topology file was generated using sobtop_1.0(dev3.1) software based on the ligand structure, with charge assignment performed via the RESP method to ensure charge distribution aligns with physicochemical properties. For the receptor protein, AMBER14SB force field parameters were employed, whilst the ligand’s molecular parameters utilized the GAFF2 force field. During simulations, the system was solvated using the TIP3P water model within a 1 nm cubic water box to ensure adequate solvation and electrical neutrality. To guarantee the system’s electrical neutrality, Na^+^ and Cl^-^ ions were introduced using GROMACS’ gmx genion tool at a concentration of 0.15 M NaCl. The charge neutralization and ionic strength conditions were set to standard physiological parameters throughout the simulation. Long-range electrostatic interactions were handled using the particle-mesh Ewald (PME) method, with a cutoff distance set at 1 nm. Force field parameters and PME method settings were optimized according to GROMACS specifications. Bond constraints were managed via the LINCS algorithm. Prior to molecular dynamics simulations, the system underwent an energy optimization process. Energy minimization comprised 3000 steps of steepest descent followed by 2000 steps of conjugate gradient optimization. The optimization was conducted in three stages: first, solute molecules were constrained while water molecules underwent minimization; subsequently, counter ions were constrained during minimization; finally, the entire system underwent unconstrained minimization. During simulation, temperature was maintained at 310 K using a Nosé–Hoover thermostat, while pressure was held at 1 bar via a Parrinello–Rahman isobaric coupler. The simulation duration was set to 100 ns, employing an NPT (isobaric-isothermal) ensemble with an integration step size of 2 fs. Throughout the simulation, GROMACS utilities gmx_rmsd, gmx_rmsf, gmx_hbond, gmx_Rg, and gmx_sasa tools to calculate the root mean square deviation (RMSD), root mean square fluctuation (RMSF), hydrogen bonds, radius of gyration (Rg), and solvent-accessible surface area (SASA), respectively, to analyze the system’s stability, structural changes, and solvent effects.

## 3 Results

### 3.1 Identification and validation of DEGs in BC

Extract datasets GSE10810, GSE33447 and GSE7904 (**Figures 1A, C**). Using the sva package, batch effects were removed from the three merged datasets (**Figures 1B, D**). Following batch normalization, a total of 1855 differentially expressed genes were identified (**Figure 1E**) and subjected to cluster analysis.

### 3.2 Identification of commonly DEGs in BC through weighted gene co-expression network analysis

**Figure 2:**
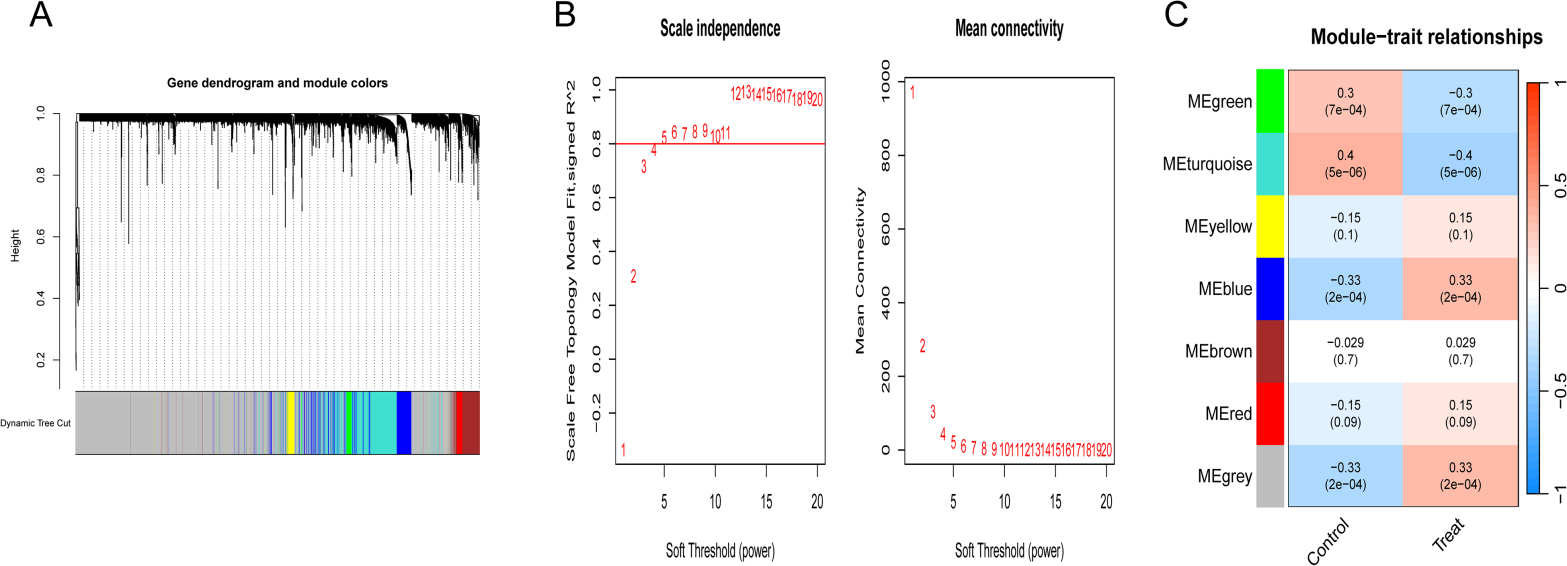
WGCNA identifies DEGs commonly observed in BC. **(A)** Scaleless plot and average connectivity plot; **(B)** Gene co-expression network map; **(C)** Module correlation heatmap.

A weighted gene co-expression network was constructed using the merged, batch-cleaned datasets GSE10810, GSE33447, and GSE7904 to identify co-expression modules and genes associated with BC. A total of seven BC co-expression modules were identified, with the MEturquoise module exhibiting the highest correlation (r = -0.4, P = 0.000005). This yielded 861 module genes (**Figure 2**).

### 3.3 GO and KEGG enrichment analysis

**Figure 3:**
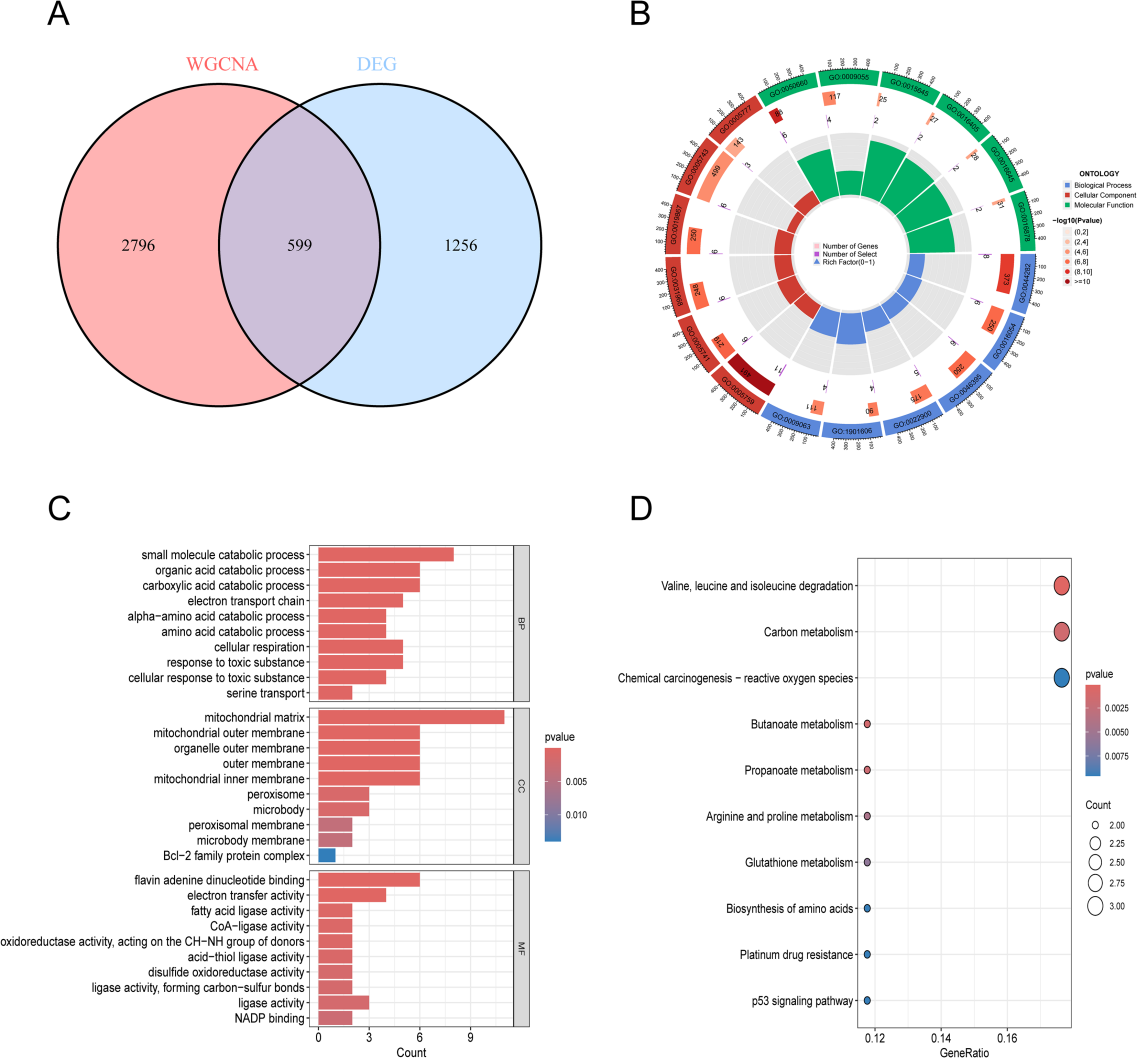
Gene enrichment analysis and PPI interaction network analysis. **(A)** Venn diagram showing the intersection of DEGs and WGCNA. **(B)** Bar chart of GO enrichment analysis. **(C)** Dendrogram of GO enrichment analysis. **(D)** KEGG enriched pathways.

The intersection of DEGs and WGCNA modules yielded 599 BC hub gene sets (**Figure 3A**). GO and KEGG analyses were conducted on the 28 final hub genes obtained by intersecting BC and mitochondrial function-related genes, This enables a comprehensive understanding of the biological functions, metabolic pathways, and interactions of similarly expressed proteins. We obtained 240 GO terms (**Figures 3B, C**) and 10 KEGG pathways (**Figure 3D**), displaying the top 10 results for each GO term by default sorting. We have identified pathways involving these proteins that are associated with the organic acid catabolic process, carboxylic acid catabolic process, mitochondrial matrix, mitochondrial outer membrane, flavin adenine dinucleotide binding, electron transfer activity, Valine, leucine and isoleucine degradation and Carbon metabolism.

### 3.4 Identifying diagnostically valuable hub genes through machine learning

**Figure 4:**
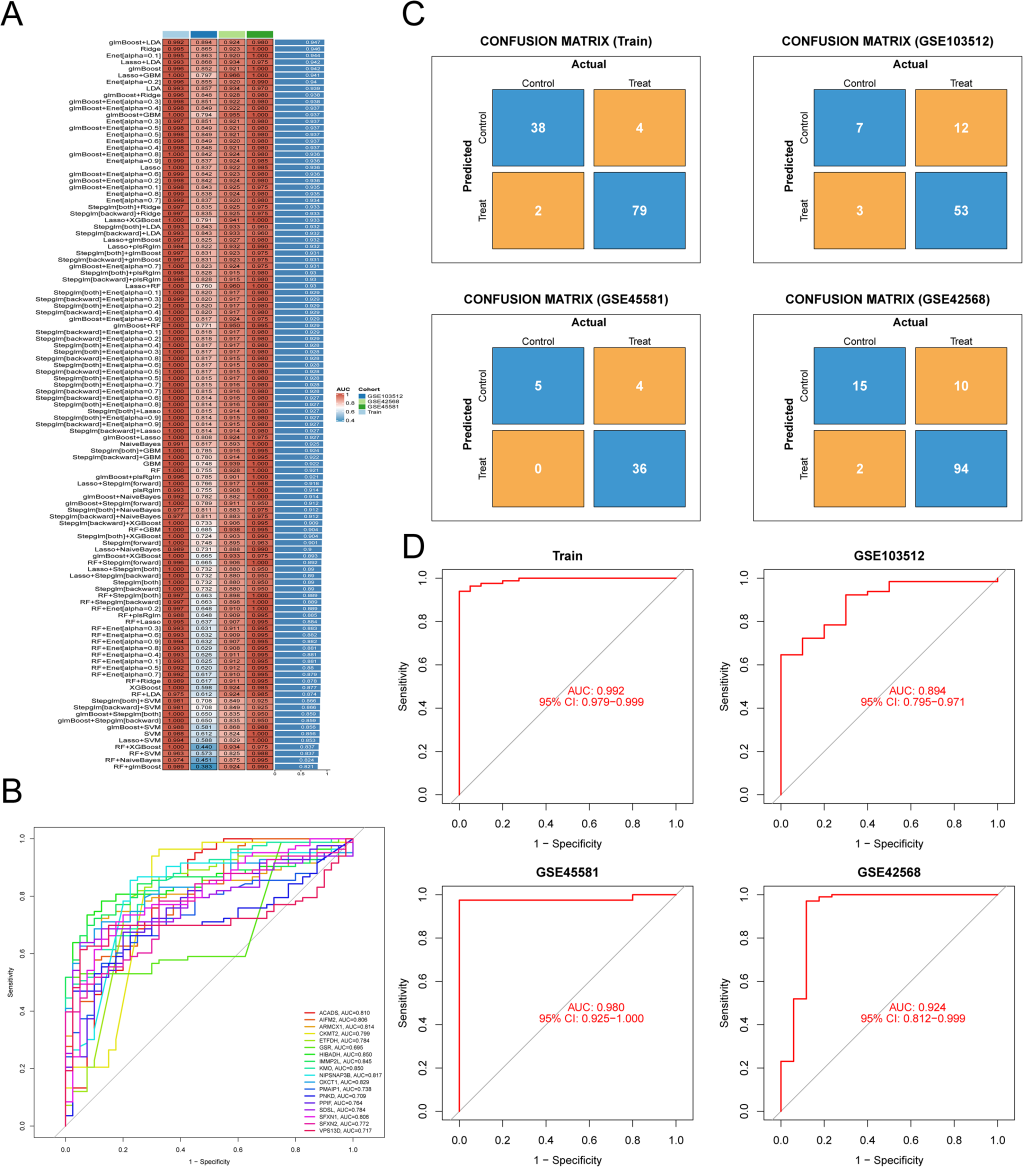
Machine learning was employed to construct diagnostic models for mitochondrial-associated BC genes. **(A)** The model constructed using a glmBoost+LDA approach demonstrated superior performance.”+”means that the second machine learning algorithm is combined on the basis of the first one. The second machine learning algorithm is classified according to the alpha value. **(B)** The diagnostic value of different hub genes in BC was evaluated via ROC analysis. **(C)** The efficacy of the model biomarkers was assessed through confusion matrices in both the training and validation cohorts. **(D)** The efficacy of the model biomarkers was evaluated via ROC analysis in both the training and validation cohorts.

Among the 113 evaluated machine learning combinations, the glmBoost+LDA model showed the highest predictive performance (C-Index = 0.947) (**Figure 4A**). This model identified 18 potential biomarkers: ACADS, AIFM2, ARMCX1, CKMT2, ETFDH, GSR, HIBADH, IMMP2L, KMO, NIPSNAP3B, OXCT1, PMAIP1, PNKD, PPIF, SDSL, SFXN1, SFXN2, and VPS13D. The diagnostic value of different hub genes in BC was evaluated using ROC curves and AUC values. It was found that ACADS (AUC = 0.810) and AIFM2 (AUC = 0.806) may have high sensitivity and specificity in BC diagnosis (**Figure 4B**). The model demonstrated strong diagnostic accuracy, with an AUC value of 0.992 (95% confidence interval [CI] 0.979-0.999) in the training cohort, 0.894 (95% CI 0.795-0.971) in the validation cohort GSE103512, 0.980 (95% CI 0.925-1.000) in GSE45581, and 0.924 (95% CI 0.812-0.999) in GSE42568 (**Figure 4D**). The confusion matrix further verified the reliability of the model, showing high sensitivity and specificity in distinguishing BC patients from the control group. In the training cohort, the sensitivity was 97.5% (79/[79 + 2]) and the specificity was 90.5% (38/[38 + 4]). In the validation cohort GSE103512, the sensitivity was 91.4% (53/[53 + 3]) and the specificity was 36.8% (7/[12 + 7]). In GSE45581, the sensitivity was 100% (36/[36 + 0]) and the specificity was 55.6% (5/[5 + 4]). In GSE42568, the sensitivity was 97.9% (94/[94 + 2]) and the specificity was 60% (15/[15 + 10]) (**Figure 4C**). Additionally, we utilized RNA-seq data from the TCGA-BRCA cohort to validate the diagnostic value of potential biomarkers and assess model performance across platforms. Results demonstrated that all 18 core genes achieved AUC values > 0.7 in the TCGA-BRCA RNA-seq dataset. The model maintained excellent performance across key metrics in the TCGA-BRCA RNA-seq data (AUC = 0.983 [95% CI 0.975-0.99], sensitivity=99.8%, specificity=54%, accuracy=95.6%) (**Supplementary Figure S1**, **Supplementary Table S3**). Collectively, these results confirm the robust predictive performance of the StepGLM+GBM model.

### 3.5 GSEA enrichment results

To further explore the functional characteristics of the key module gene sets, we conducted GSEA enrichment analysis and visualized the results (**Supplementary Figure S2**). Analysis revealed that the key gene GSR exhibited significant enrichment across multiple pathways. The KEGG pathways enriched in the high-expression group included Amino sugar and nucleotide sugar metabolism and Sphingolipid metabolism; whereas the low-expression group featured Complement and coagulation cascades and Cytokine-Cytokine Receptor Interaction. Similarly, the ACADS high-expression group was predominantly enriched in Drug metabolism - Cytochrome P450 and the PPAR signaling pathway; the low-expression group concentrated in Antigen processing and presentation and Riboflavin metabolism. Analysis indicates that the high- and low-expression groups within key modules respectively exhibit enrichment in distinct KEGG pathways. These findings provide crucial insights into the molecular mechanisms and functional characteristics of mitochondrial-associated BC, while offering reference points for identifying potential therapeutic targets.

### 3.6 Immunohistochemical analysis

**Figure 5:**
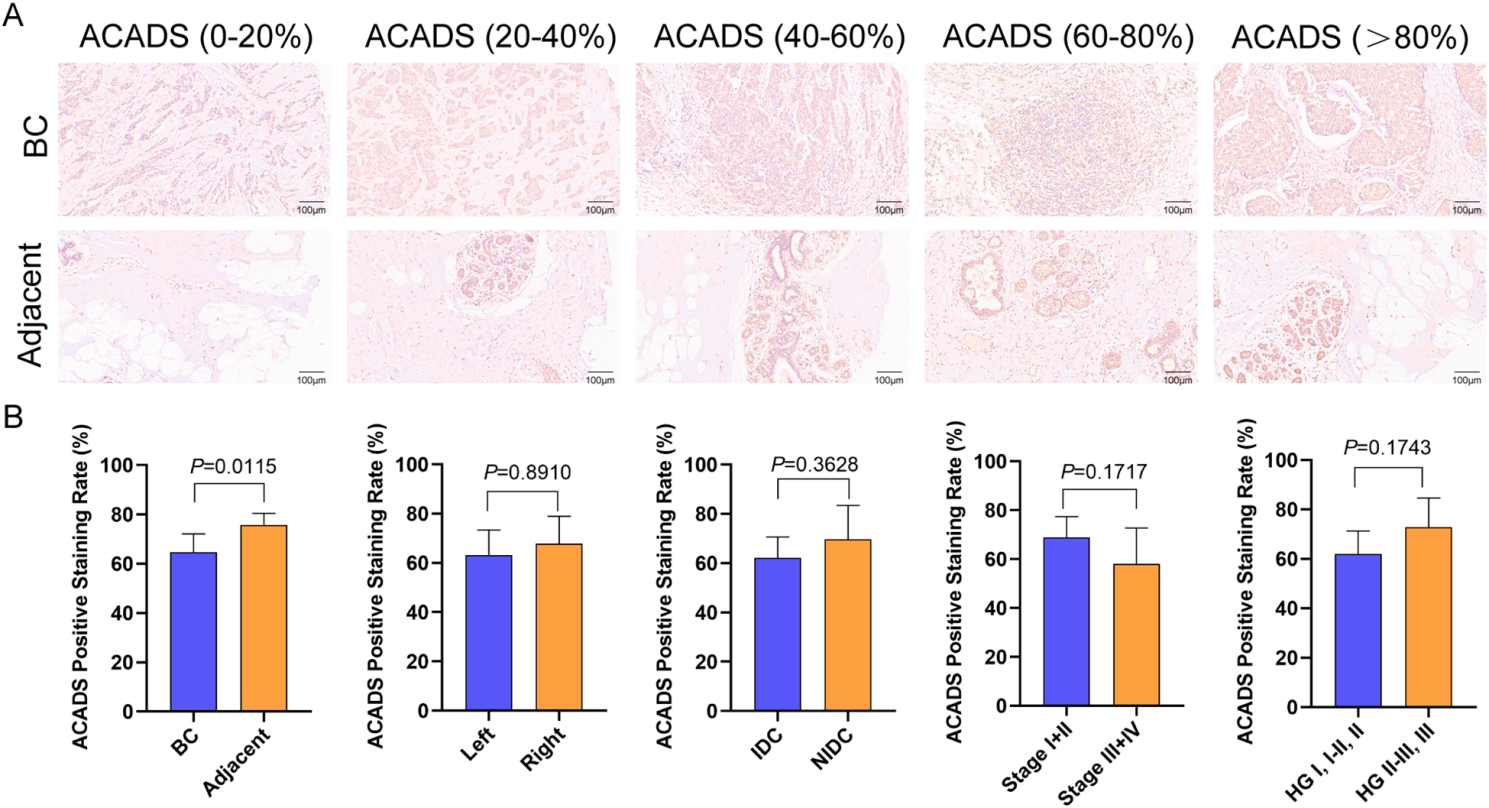
High-throughput validation of ACADS expression in BC tissue chip. **(A)** Analysis of ACADS expression in BC versus adjacent tissues using IHC. **(B)** Evaluation of ACADS expression relative to various clinicopathological parameters.

To validate the protein expression levels of key genes in BC, we analyzed immunohistochemical results from the HPA database. Representative immunohistochemical images further confirmed that the protein expression levels of ACADS, ARMCX1, ETFDH, HIBADH, IMMP2L, NIPSNAP3B, and OXCT1 were significantly downregulated in BC tissue, whereas GSR, SDSL, SFXN1, and SFXN2 were upregulated. These findings suggest the potential value of key proteins as therapeutic targets for BC (**Supplementary Figure S3**). Further investigation of ACADS expression utilized a high-throughput BC tissue microarray (Catalogue No.: HBreD120CS01), revealed significantly lower expression scores in cancerous tissue compared to adjacent non-cancerous tissue (*P=*0.0115). However, no significant differences were observed based on location (left vs. right), cancer subtype (IDC vs. NIDC), clinical stage (I+II vs. III+IV), or histologic grading (I, I-II, II vs. II-III, III) (**Figure 5**).

### 3.7 Immune infiltration analysis

**Figure 6:**
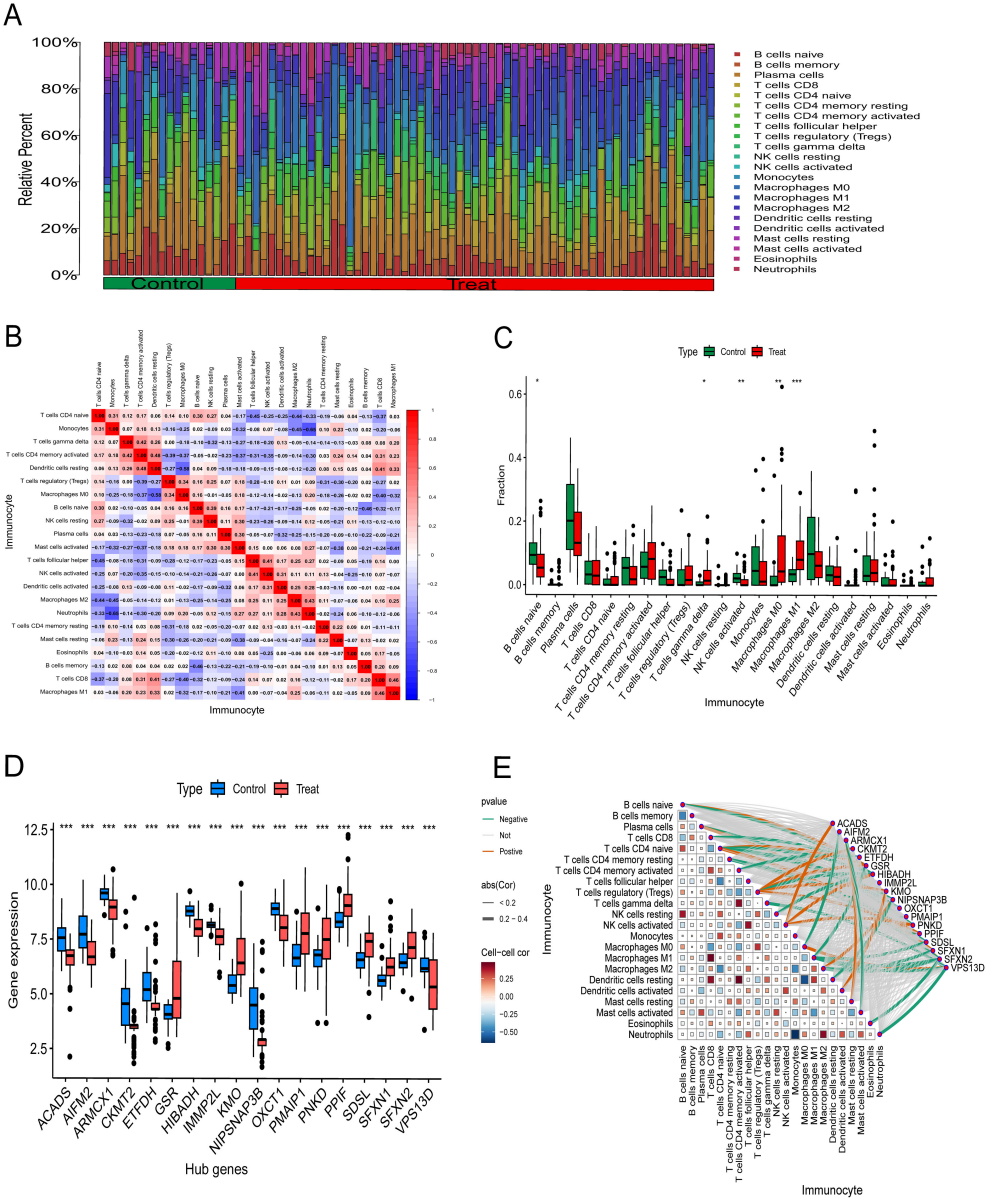
Analysis of immune cell infiltration of key genes. **(A)**Superimposed histograms show the proportion of immune cells in the experimental and control groups. **(B)** The heatmap illustrates the correlation between model hub genes and the infiltration of 22 immune cell types. **(C)** Box plots comparing 22 immune cell types between the experimental and control groups. *p < 0.05; **p < 0.01; ***p < 0.0001; ns, not significant. Immunological correlation results presented. **(D)** Distribution of expression levels for pivotal genes in the control group versus the experimental group. **(E)** Positive and negative regulatory relationships between genes and immune cells, where brown denotes positive correlation and green denotes negative correlation.

Using the CIBERSORT algorithm, we assessed differences in immune cell infiltration across 22 immune cell subtypes between the BC experimental group and the control group. Based on the immune infiltration analysis results, we generated an overlay histogram to display the relative proportions of the 22 immune cell subtypes within each sample (**Figure 6A**). It is noteworthy that correlation analysis (**Figure 6B**) revealed a significant negative correlation between Monocytes and Neutrophils (r = −0.65), whilst resting Dendritic cells exhibited a positive correlation with activated CD4 memory T cells (r = 0.48).Additionally, we employed box plots to compare the distribution differences of each immune cell subtype between the experimental and control groups. Compared with the control group, significant differences were observed in naive B cells, gamma delta T cells, activated NK cells, M0 macrophages, and M1 macrophages. These differentially expressed cell subpopulations may participate in regulating tumor immune responses in BC, providing direction for further exploration of immune regulatory characteristics within the BC microenvironment (**Figure 6C**).

### 3.8 Hub genes and immune cell relevance

Box plot analysis revealed significant differences in the expression level distribution of 18 key genes between the BC control group (Control) and the experimental group (Treat) (**Figure 6D**). Based on the correlation analysis results between these 18 model key genes and 22 immune cell subtypes, we further discovered that ACADS exhibits a highly positive correlation with activated NK cells, while AIFM2 shows a significant negative correlation with M0 macrophages. It was also observed that GSR was positively correlated with B cells naive, T cells CD4 naive, and T cells regulatory (Tregs), and negatively correlated with T cells CD8 and Macrophages M1. HIBADH was negatively correlated with B cells naive, T cells CD4 naive, T cells regulatory, NK cells resting negatively and positively correlated with NK cells activated (**Figure 6E**).

### 3.9 Single-cell cluster analysis of key genes

**Figure 7:**
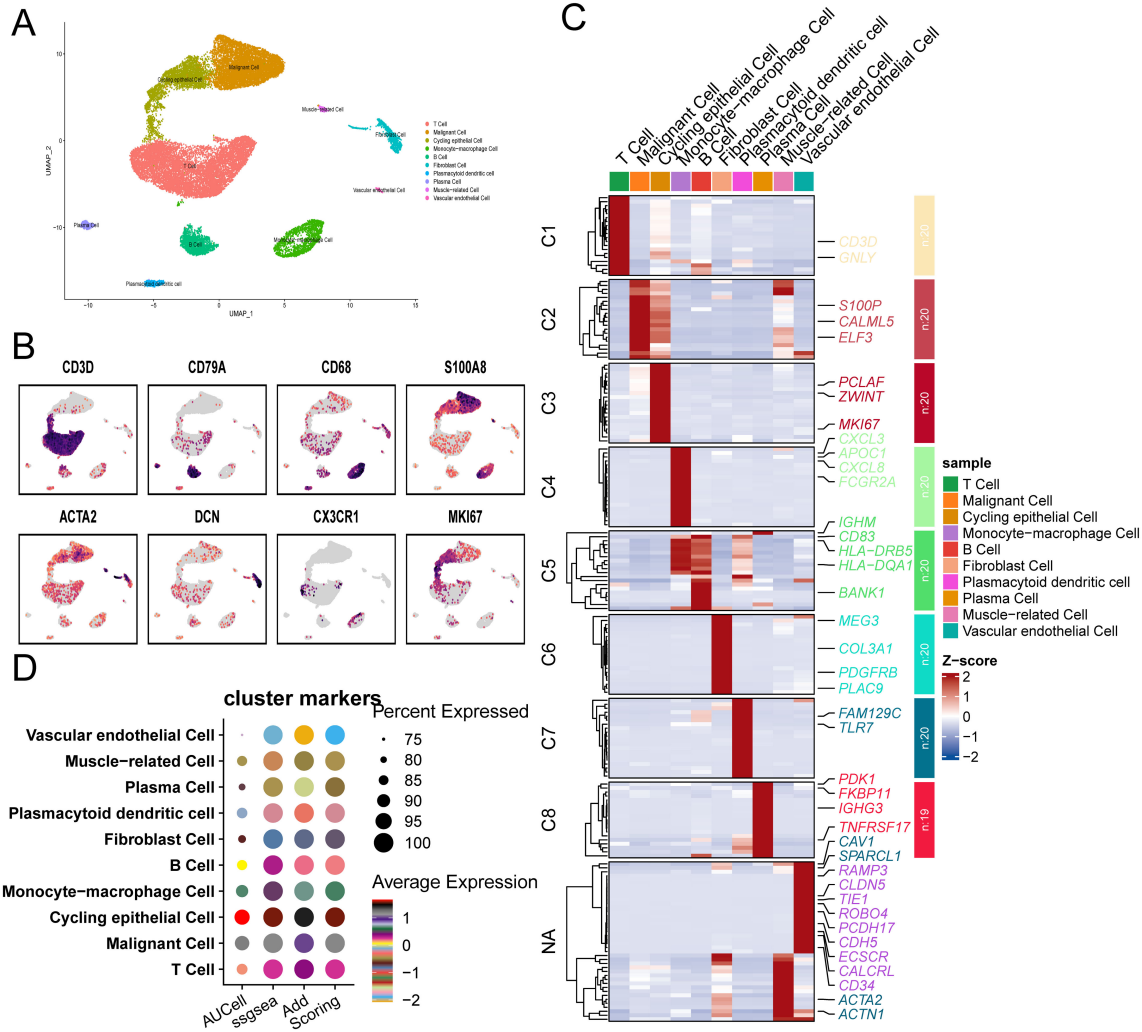
Single-cell cluster analysis of key genes. **(A)** Gene expression based visualization of cell clusters identifying different cell types in the tumor microenvironment such as T cell, monocyte-macrophage cell and malignant cell. **(B)** The spatial distribution of specific marker genes in different cell types provides insights into their role and location in the tissue. **(C)** Displays gene expression levels across distinct cell clusters, highlighting differentially expressed genes potentially involved in BC biology. **(D)** Presents the average expression and percentage of cells expressing cluster-marker genes, summarizing cell-type-specific expression patterns.

The UMAP plot shows single-cell clustering based on their gene expression profiles. Different cell types are color-coded, including T cell, cycling epithelial cell, malignant cell, monocyte-macrophage cell, B cell, muscle-related cell, etc. This visualization helps to identify different cell clusters in the dataset (**Figure 7A**). Gene expression profiles showing the expression levels of specific marker genes (CD3D, CD79A, CD68, S100A8, ACTA2, DCN, CX3CR1 and MI67) across the UMAP cluster. Color intensities represent expression levels, highlighting the distribution of these markers in different cell types (**Figure 7B**). Heatmap showing the expression levels of selected genes in different cell clusters (C1-C7). Each row represents a gene and each column represents a cell cluster. The color gradient indicates the expression level, with red representing high expression and blue representing low expression. The dendrogram on the left clusters genes with similar expression patterns (**Figure 7C**). The dot plot illustrates the average expression and percentage of cells expressing specific marker genes in different cell types (**Figure 7D**).

### 3.10 Gene expression analysis across different cell types in a BC context

**Figure 8:**
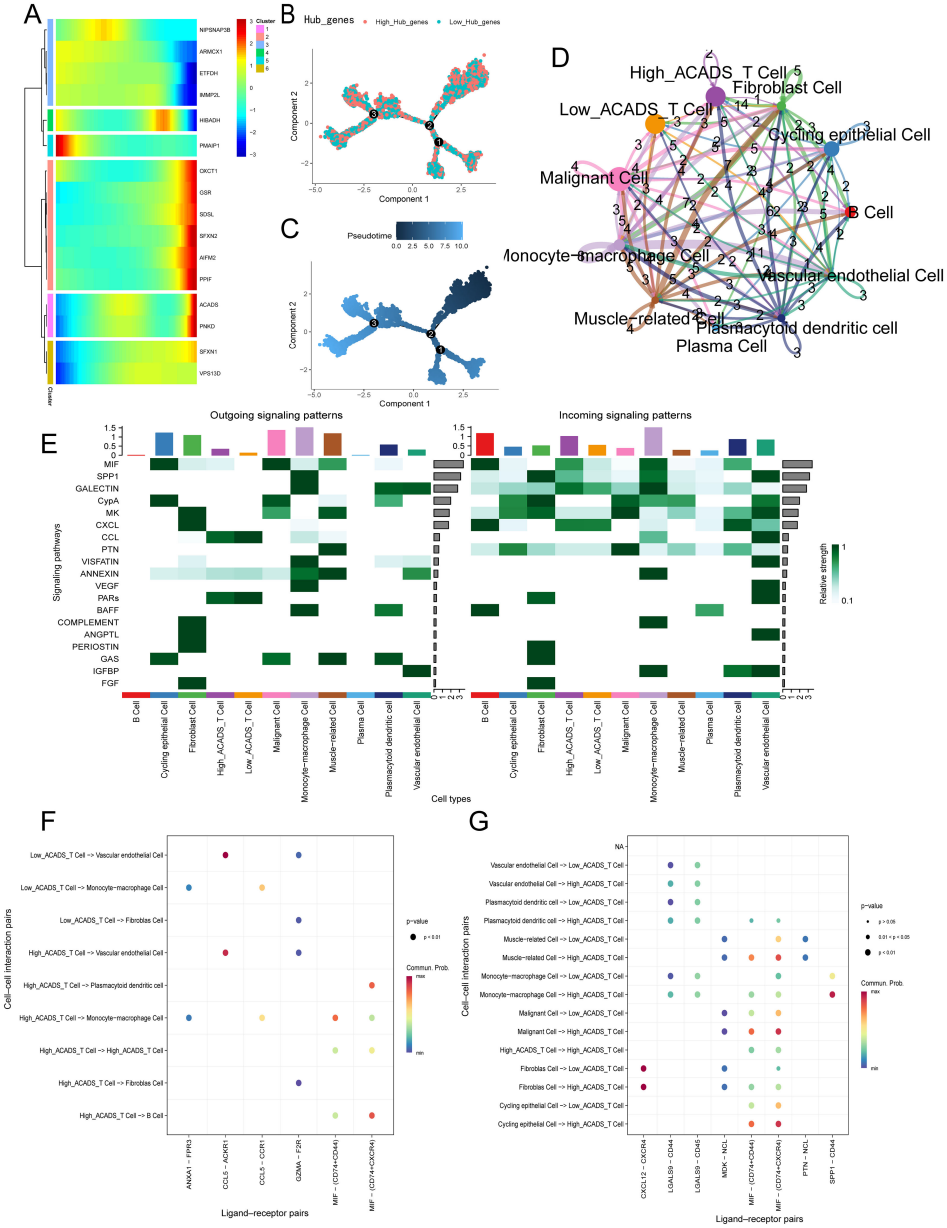
Gene expression analysis of different cell types in BC context. **(A)** Heatmap of hub genes expression during cellular lineage progression. **(B)** Monocle's inferred developmental trajectory scatter plot. **(C)** Pseudotemporal trajectories indicate changes in gene expression over time. **(D)** Network diagram of interactions between T cells with different ACADS gene expression profiles and various cell types. **(E)** A heatmap showing the outgoing and incoming signaling patterns of different cell types. The horizontal axis represents different cell populations or subtypes, the vertical axis represents signaling pathways, and the color intensity indicates the signal strength. **(F)** Each dot represents the contribution strength of a specific signaling pathway between T cells with high/low ACADS expression and other cell populations. The horizontal axis and vertical axis represent the signaling sender and receiver cell populations, respectively. **(G)** Dot plot of the interaction between incoming signals and T cells with high/low ACADS expression.

Heatmap of hub genes expression during cellular lineage progression (**Figure 8A**). Monocle's inferred developmental trajectory scatter plot (**Figure 8B**). Pseudotemporal maps illustrate the progression of cell states along developmental trajectories. The color gradient indicates pseudotemporal, providing insights into the dynamics of gene expression as the cell transitions through different states (**Figure 8C**). This network diagram represents the interactions between T cells with different ACADS gene expression profiles and various cell types, such as cycling epithelial cell, monocyte-macrophage cell, B cell and other cells. The size of the lines indicates the strength of the interactions, highlighting the key communication pathways through which ACADS exerts its effects in the tumor microenvironment (**Figure 8D**). This heatmap illustrates the outgoing and incoming signaling patterns of different cell types. Each row represents a signaling pathway (e.g., MIF, CypA, and SPP1 signaling pathways), and each column represents a distinct cell type. The depth of color indicates the relative strength of signal transduction, providing a reference basis for analyzing intercellular communication (**Figure 8E**). This dot plot visualizes the outgoing signaling interactions from T cells with high/low ACADS expression to other cell types. The size of the dots indicates the importance of the interactions, while the color represents the strength of the signaling pathways (**Figure 8F**). Similar to Panel F, this dot plot shows the incoming signaling interactions from other cell types to T cells with high/low ACADS expression. The size of the dots reflects the importance, and the color represents the signal strength (**Figure 8G**).

### 3.11 Drug forecasting analysis

**Figure 9:**
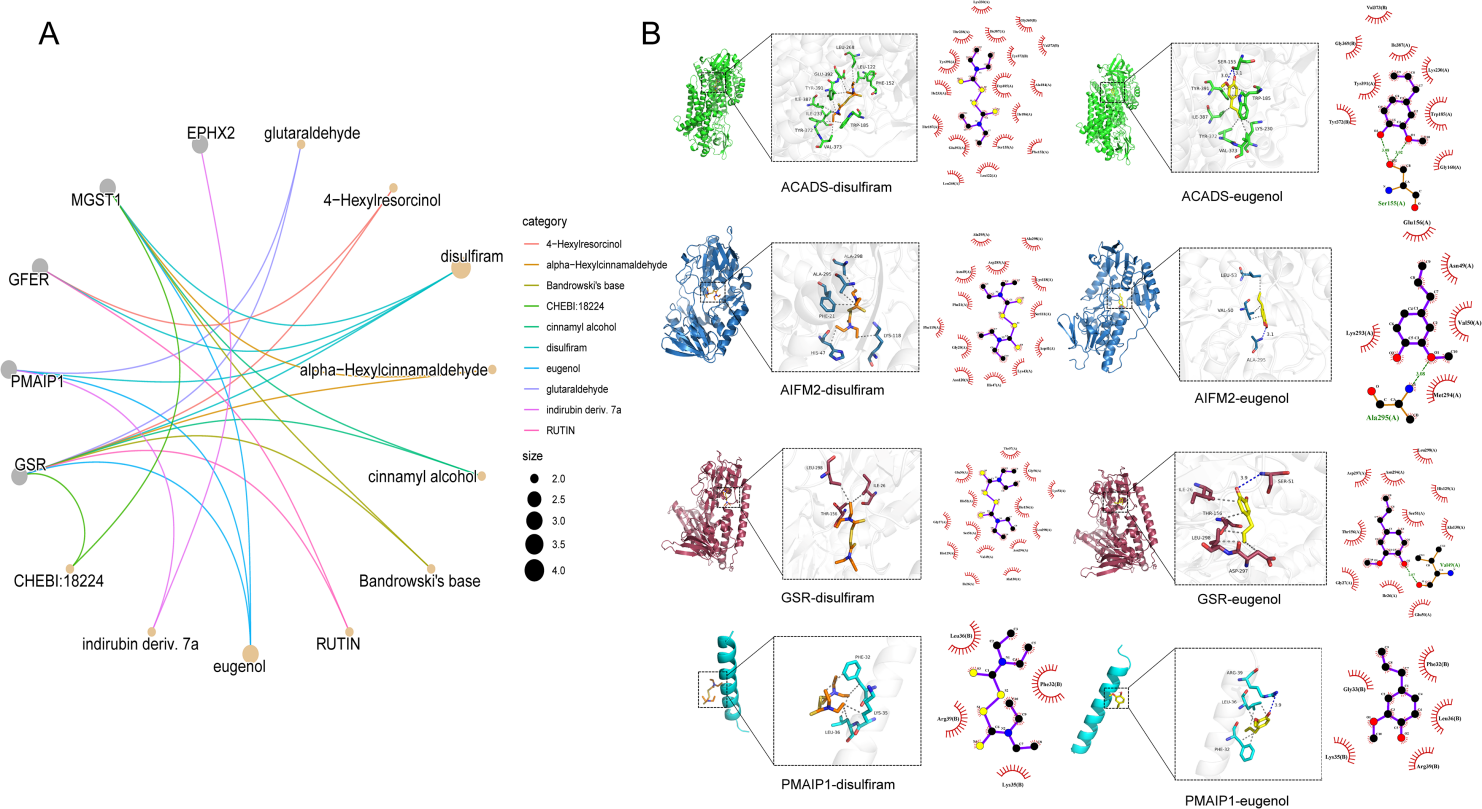
Results of target-drug enrichment and molecular docking. **(A)** Target-drug enrichment results. The grey nodes in the figure represent genes, the orange nodes represent drugs, and the connecting lines represent drug prediction relationships. **(B)** Molecular docking demonstration of partial drugs and targets.

Since most drugs exert their therapeutic effects by targeting proteins, we combined drug-gene interaction analyses to screen disulfiram and eugenol as potentially effective drugs for BC, whereas GSR and PMAIP1 were found to be associated with a variety of drugs and may be important targets for the treatment of BC (**Figure 9A**).

### 3.12 Molecular docking

In order to explore the molecular mechanisms predicting the therapeutic effects of the drugs on BC targeting, molecular docking experiments of the key drugs disulfiram and eugenol with the pivotal genes ACADS, AIFM2, GSR, and PMAIP1 were performed in this study. Generally, a binding energy below 0 indicates that proteins and small molecules can spontaneously bind; below -5 kcal·mol^-^¹ signifies stable binding. A lower binding energy indicates stronger binding between the ligand and receptor. The docking results were visualized in three dimensions using PyMOL 2.3.0 software, and analyzed in two dimensions using LigPlot v.2.3(**Figure 9B**). The molecular docking results showed that the predicted drugs could form stable conformations with the hub genes (**Table 1**).

**Table 1:** Molecular docking results of partial drugs and targets.

Protein	Compound	Center (x, y, z)	Grid Box Size (x, y, z)	Binding Energy (kcal·mol^-1^)
GSR	Disulfiram	60.0, -18.3, 51.7	20,20,20	-5.0
PMAIP1	Disulfiram	9.5, -24.3, 16.4	20,20,20	-2.8
ACADS	Disulfiram	-9.9, -10.0, 19.7	20,20,20	-6.0
AIFM2	Disulfiram	-25.0, -13.4, 20.1	20,20,20	-5.3
GSR	Eugenol	60.0, -18.3, 51.7	20,20,20	-5.9
PMAIP1	Eugenol	9.5, -24.3, 16.4	20,20,20	-4.0
ACADS	Eugenol	-9.9, -10.0, 19.7	20,20,20	-6.9
AIFM2	Eugenol	-25.0, -13.4, 20.1	20,20,20	-6.2

Experimental results indicate that ACADS and AIFM2 exhibit the lowest binding energies with eugenol, at -6.9 kcal·mol^-^¹ and -6.2 kcal·mol^-^¹ respectively, demonstrating optimal binding energy. Three-dimensional and two-dimensional visualization analyses reveal that eugenol interacts with the target protein ACADS through hydrogen bonds (blue dashed lines) and hydrophobic interactions (cilium-like structures). Further examination of the three-dimensional visualization shows that eugenol forms two hydrogen bonds with the amino acid residue SER155 in ACADS, at distances of 3.0 and 3.1 Å respectively. The 2D analysis reveals that eugenol interacts with the target protein residues GLY369, GLY160, TRP185, TYR391, ILE387, TYR372, VAL373, and LYS230. Furthermore, docking results indicate a binding energy of -6.9 kcal·mol^-^¹, demonstrating that eugenol spontaneously binds to the receptor protein with stable interaction. Consequently, it is highly likely to exert pharmacological effects. Eugenol forms one hydrogen bond with amino acid residue ALA295 in target protein AIFM2 at a distance of 3.1 Å. 2D analysis reveals hydrophobic interactions between eugenol and target protein residues GLU156, ASN49, VAL50, MET294, and LYS293, with a binding energy of -6.2 kcal·mol^-^¹. The interaction types are summarized in **Table 2**.

**Table 2:** Types of protein-ligand interactions.

Protein	Compound	H-Bond	Hydrophobic interaction
GSR	Disulfiram	0	14
PMAIP1	Disulfiram	0	4
ACADS	Disulfiram	0	17
AIFM2	Disulfiram	0	13
GSR	Eugenol	1	10
PMAIP1	Eugenol	1	5
ACADS	Eugenol	2	8
AIFM2	Eugenol	1	5

### 3.13 Molecular dynamics simulation of protein-ligand complexes

We selected the protein-ligand pairs with the optimal molecular docking binding energy (ACADS-Eugenol and AIFM2-Eugenol) and conducted 100 ns molecular dynamics simulations.

**Figure 10:**
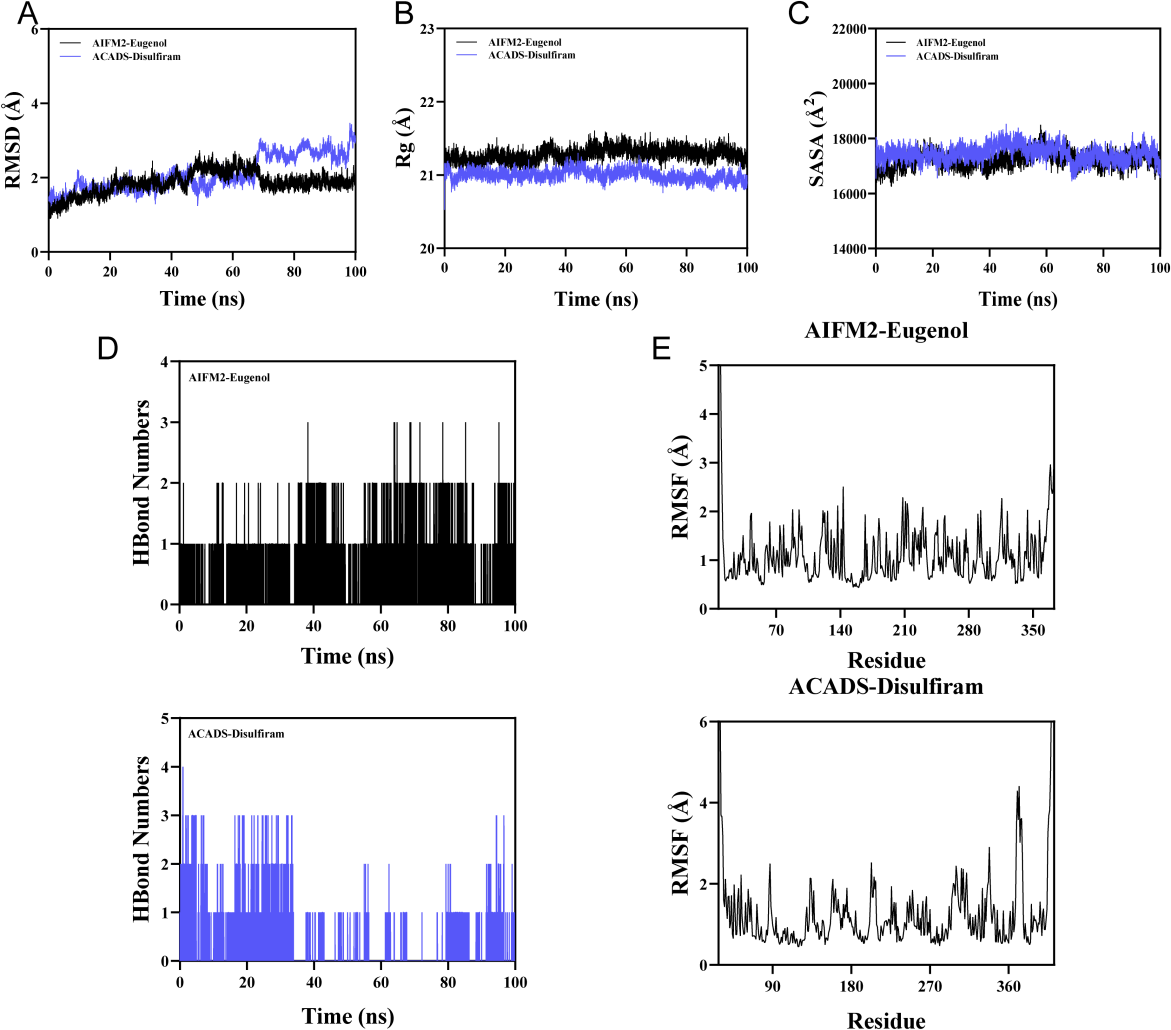
Molecular dynamics simulation of the protein-ligand complex. **(A)** RMSD values of the protein-ligand complex over time. **(B)** Rg values of the protein-ligand complex over time. **(C)** SASA values of the protein-ligand complex over time. **(D)** HBonds values of the protein-ligand complex over time. **(E)** RMSF values of the protein-ligand complex.

RMSD serves as a reliable metric for assessing the conformational stability of proteins and ligands, as well as measuring the degree of deviation in atomic positions from their initial locations. A smaller deviation indicates greater conformational stability. Therefore, RMSD was employed to evaluate the equilibrium of the simulated systems. As shown in **Figure 10A**, the AIFM2-Eugenol complex system reached equilibrium after 70 ns, ultimately fluctuating around 2 Å. The ACADS-eugenol complex exhibits stable fluctuations between 10–50 ns, displaying a slight upward trend in the late motion phase while consistently fluctuating below 3 Å. Consequently, the eugenol small molecule achieves relatively stable binding with the AIFM2 and ACADS target proteins.

Rg characterizes overall structural changes and reflects protein packing density. Both AIFM2-Eugenol and ACADS-Eugenol complexes exhibited stable fluctuations during motion, indicating no significant expansion or contraction of the small molecule-target protein complexes during movement (**Figure 10B**).

SASA serves as an indicator for evaluating protein surface area. Simulations calculated the SASA between the target protein and small molecules (**Figure 10C**). Results showed no significant changes in the SASA of the AIFM2-Eugenol and ACADS-Eugenol complexes after ligand binding, indicating minimal structural impact of ligand association on the proteins.

Hydrogen bonds play a crucial role in ligand-protein interactions. The number of hydrogen bonds between the small molecule and the target protein during the kinetic process is shown in **Figure 10D**. The number of hydrogen bonds between the AIFM2-Eugenol small molecule and the target protein ranged from 0 to 3, with most complexes exhibiting approximately 2 hydrogen bonds. The number of hydrogen bonds between the ACADS-Eugenol small molecule and the target protein ranges from 0 to 4, with most complexes exhibiting approximately one hydrogen bond. This indicates that this ligand exhibits favorable hydrogen bonding interactions with the target protein.

RMSF quantifies the flexibility of amino acid residues within a protein. As shown in **Figure 10E**, the RMSF values for the AIFM2-Eugenol complex are relatively low (mostly below 2.1 Å), while those for the ACADS-Eugenol complex are also relatively low (mostly below 4 Å). Consequently, both complexes exhibit low flexibility and high stability.

In summary, the AIFM2-Eugenol and ACADS-Eugenol complex systems exhibit stable binding with well-developed hydrogen bonding interactions. Consequently, the Eugenol small molecule demonstrates favorable binding energy with both AIFM2 and ACADS target proteins.

## 4 Discussion

BC is one of the most common malignant tumors among women worldwide, with its high degree of heterogeneity and drug resistance posing significant treatment challenges (27). It has been established that mitochondrial dysfunction correlates with BC progression. Research indicates that inhibiting ATP synthase activity within the mitochondrial matrix promotes glycolysis (the Warburg effect), suggesting that reprogramming of mitochondrial matrix energy metabolism may influence BC tumor metastasis by regulating extracellular matrix integrity (28). The dynamic equilibrium of mitochondrial fusion/fission is also closely linked to BC, influencing apoptotic signaling by regulating mitochondrial outer membrane permeability. Altering the mitochondrial network structure can affect the metabolic adaptability and drug resistance of BC tumor cells (29–31). This study aims to develop a reliable computational model capable of distinguishing between tumor and normal tissue by integrating multiple machine learning approaches based on mitochondrial-related gene biomarkers, thereby aiding in BC diagnosis. Key gene expression will be experimentally validated, with the objective of exploring novel directions for personalized targeted therapy in clinical practice through analysis of key genes and immunoregulatory mechanisms.

We obtained 28 final hub genes by taking the intersection of BC-related genes and mitochondrial function-related genes, and conducted GO and KEGG enrichment analyses on these genes. Ultimately, we obtained 240 GO terms. The BP primarily encompassed processes such as organic acid catabolic process and carboxylic acid catabolic process, whilst the CC included mitochondrial matrix and mitochondrial outer membrane. MF included flavin adenine dinucleotide binding and electron transfer activity. Lactic acid, as a core metabolite in organic acid catabolism, enhances lactate shuttling. This process promotes vascularization, immune evasion, and cellular invasion in BC cells by acidifying the microenvironment (32). Acetate metabolism constitutes a pivotal branch of the carboxylic acid catabolic pathway. By converting acetate into acetyl-CoA, it enables energy provision to BC cells via the tricarboxylic acid cycle. Promoting acetate metabolism enhances T-cell effector function and antitumor immunity (33).

Based on the key metabolic processes revealed by GO analysis, we further explored the functional roles of these genes at the pathway level through KEGG enrichment analysis. KEGG enrichment analysis yielded ten pathways, primarily involving valine, leucine and isoleucine degradation, butanoate metabolism, and carbon metabolism. Research by Pei-Jing Yang et al. revealed that in the plasma metabolomics of recurrent BC patients, the degradation pathways of valine, leucine, and isoleucine were significantly enriched. Furthermore, elevated valine levels were found to be significantly negatively correlated with recurrent BC compared to low plasma valine levels (34). Butyrate induces apoptosis in colorectal cancer cells by enhancing the hyperactivation of the Wnt signaling pathway (35). Although abnormal activation of the Wnt/β-catenin pathway is a common feature of BC, the mechanism of action of butyrate on BC remains unclear^[20]^.Research demonstrates that B vitamins (folate, B_2_, B_6_, B_12_) within the one-carbon metabolism (1CM) pathway significantly influence BC occurrence and prognosis by regulating DNA synthesis and methylation processes. Concurrently, 1CM-associated genes such as MTHFR and MTR exhibit high expression in breast tissue, participating in oxidative stress and epigenetic regulation (36), synergistically promoting BC tumor growth alongside the PI3K/Akt signaling pathway (37). These pathways and associated genes provide potential targets for the screening of diagnostic biomarkers and mechanistic studies in BC.

Subsequently, we constructed a BC diagnostic model based on the glmBoost+LDA approach by integrating 113 machine learning algorithms. This model demonstrated robust diagnostic efficacy and generalizability across multiple datasets. Concurrently, we identified 18 diagnostically valuable mitochondrial-associated gene biomarkers, including ACADS, AIFM2, ARMCX1, GSR, and PMAIP1, and analyzed their functional roles via GESA. Furthermore, we conducted experimental validation and functional exploration of the core genes. To validate ACADS expression in clinical BC, we employed high-throughput tissue chip combined with IHC to analyze 56 BC and adjacent non-cancerous tissue samples. Consistent with our findings, ACADS expression scores were significantly lower in cancerous tissue than in adjacent non-cancerous tissue (P = 0.0115), demonstrating ACADS’s potential diagnostic value in BC. DNA methylation of ACADS has been established as a key regulatory mechanism governing proliferation and metastasis in hepatocellular carcinoma (38). However, the potential association with BC remains unclear at present. Nevertheless, based on shared epigenetic regulatory mechanisms, ACADS methylation may hold exploratory value in BC metabolic reprogramming or metastasis. GESA analysis revealed that the ACADS high-expression group was predominantly enriched in Drug metabolism - Cytochrome P450 and the PPAR signaling pathway, whilst the low-expression group concentrated in Antigen processing and presentation and Riboflavin metabolism. The CYP pathway primarily mediates the metabolism of anticancer drugs, enhancing drug tumor specificity whilst reducing toxicity to normal tissues, thereby targeting the inhibition of BC cell proliferation (39). CYP450 influences disease progression in BC through multiple mechanisms, including mediating chemotherapy drug metabolism (40) and regulating tumor endogenous metabolism (41). It may serve as a prodrug activator for selectively killing ER^+^BC cells overexpressing CYP1 (42). The PPAR signaling pathway plays a crucial role in BC progression (43). PPARγ exerts tumor-suppressive effects by inhibiting proliferation and inducing differentiation. In ER^+^BC, PPARγ forms a complex with ERα, antagonizing the PI3K/AKT survival pathway, suppressing aromatase expression, and reducing local estrogen synthesis, thereby inhibiting tumor growth (44).Riboflavin plays a pivotal role in cellular energy metabolism. Research has revealed significantly elevated levels of riboflavin carrier protein (RCP) in BC patients, suggesting RCP may serve as a potential biomarker for BC (45). Furthermore, riboflavin transporter is markedly overexpressed in BC cells, potentially promoting tumor metabolic adaptation by enhancing riboflavin uptake (46). Research has revealed that overexpression of UGCG (UDP-glucose ceramide glucosyltransferase) in BC cells enhances cellular resistance to oxidative stress by upregulating GSR mRNA expression. Concurrently, it promotes glutamine metabolism to sustain proliferative advantage, suggesting that GSR may play a pivotal role in tumor metabolic adaptation (47). Furthermore, among BC patients undergoing chemotherapy, the gene expression levels of GSR have been identified as a potential biomarker for treatment response, with alterations potentially influencing drug efficacy and resistance (48). The expression of GSR may also correlate with lymph node metastasis in TNBC. Jiayue Luo et al., based on whole-transcriptome sequencing analysis, found that GSR may inhibit metastasis by regulating oxidative stress pathways, potentially serving as a significant prognostic biomarker for TNBC (49). Research has revealed that within the KEGG pathways enriched in both the GSR high-expression and low-expression groups, intervention in amino sugar and nucleotide sugar metabolism pathways inhibits DNA/RNA synthesis while promoting oxidative stress, thereby inducing cancer cell apoptosis (50). Sphingolipid metabolism exhibits significant regulatory associations with signaling pathways such as PI3K-mTORC1 and Hippo. YAP phosphorylation and activation of the sphingolipid metabolic enzyme SGPP2 jointly mediate tumor metabolic adaptation (51); whilst the sphingolipid metabolic enzyme CERK promotes drug resistance in ER+ BC (52–54). Concurrently, studies reveal that the sphingolipid metabolic enzyme PAQR4 is highly expressed in BC and mediates anti-apoptotic effects (55). Congcong Gong et al. experimentally demonstrated that the complement and coagulation cascades pathways may promote tumor progression by regulating inflammatory responses and immune evasion within the tumor microenvironment (56). Cytokine-cytokine receptor interactions influence BC progression through multiple pathways. For instance, the IL6ST family regulates proliferation and therapeutic response in ERα+ BC via the STAT/ERK pathway (57, 58), whilst IL-4 may promote malignant phenotypes (59). In BC, the mechanism of action of AIFM2 exhibits subtype-specific differences, with its overexpression promoting tumor cell proliferation, invasion and migration (60); Genome-wide analysis indicates that upregulation of AIFM2 affects BC cell proliferation and patient prognosis (61). PMAIP1 is a key regulator of the intrinsic apoptosis pathway. Research has demonstrated that the vitamin E analogue α-TEA significantly upregulates PMAIP1 by activating the JNK/p73 signaling axis, thereby inducing apoptosis in MDA-MB-435BC cells (62). In TP53 mutant BC, the regulation of PMAIP1 exhibits complexity. Subhasree Rajaram et al. demonstrated that arsenic trioxide suppresses TNBC cell proliferation by restoring mutant p53 function and activating classical p53 target genes, including PMAIP1 (63). Concurrently, investigations into the miR-644a/CTBP1/p53 axis revealed that downregulation of CTBP1 enhances the transcriptional activation of PMAIP1 by mutant p53, thereby propelling cells from cycle arrest towards apoptosis (64). Furthermore, multi-omics analysis indicates that PMAIP1 influences the tumor immune microenvironment by regulating glucose metabolism. A prognostic model constructed based on PMAIP1 and other genes can effectively distinguish BC clinical subtypes (65). The aforementioned findings suggest that these key genes may influence BC progression by regulating the tumor immune microenvironment.

To validate this hypothesis, we further analyzed the association between hub genes and immune cell infiltration. Correlation analysis between pivotal genes and immune cell types demonstrated that ACADS exhibits a highly positive correlation with activated NK cells. In BC, NK cell activation suppresses tumor growth and metastasis by enhancing immune surveillance, directly killing tumor cells (66), and modulating the tumor microenvironment (67). Downregulation of ACADS in breast tissue may lead to reduced NK cells, resulting in poor prognosis. Research has demonstrated that the formation of pulmonary metastases in primary BC can be suppressed through the synergistic action of monocytes and neutrophils (68). Meanwhile, Sheri A C McDowell et al. discovered that obesity alters the developmental trajectory of monocytes, promoting the release of neutrophil extracellular traps (NETs) and accelerating lung metastasis (69). Concurrently, neutrophil-derived Neutrophil gelatinase-associated lipocalin may support tumor growth via iron metabolism pathways (70). It is noteworthy that the dynamic equilibrium between resting dendritic cells and CD4+ T cells may influence therapeutic response. In tumors exhibiting high MMP-11 expression, concurrent reductions in both resting dendritic cells and CD4+ T cell infiltration create an immunosuppressive microenvironment, resulting in significantly diminished patient survival rates (71). High levels of infiltration by B cell naives, M1 macrophages, CD4+ T cells, and CD8+ T cells within tumor tissue constitute a potential indicator of favorable prognosis in BC patients (72, 73). High levels of M0 macrophage infiltration are significantly associated with poor prognosis in BC patients (74). Highly infiltrated M0 macrophages can increase extracellular matrix porosity by secreting pro-inflammatory factors such as L-8 and MMP9, thereby promoting tumor cell invasion and vascular extravasation (75). Reduced expression of LDHB (lactate dehydrogenase B) in BC cells promotes lactate clearance, leading to increased NK cells activity and inhibition of tumor metastasis (76). Resting NK cells are associated with functional suppression and tumor immune evasion. Research indicates that BC cells can evade immune surveillance by inducing NK cells to enter a resting state. These resting NK cells not only lose their cytotoxic function but also secrete the pro-metastatic factor TGF-β, thereby promoting the formation of metastatic foci (77). Gamma-delta T cells can improve prognosis by exerting selective cytotoxic effects through recognition of phosphoantigens overexpressed by BC tumor cells (78, 79). Tregs promote immunosuppression and tumor progression in BC through multiple mechanisms. Research indicates that Tregs undergo significant expansion within the tumor microenvironment and suppress the antitumor responses of effector T cells by recognizing breast tissue-specific antigens (80). Huimin Jiang et al. discovered that GSR expression levels correlate significantly with the degree of infiltration by CD8+ T cells, Tregs, and NK cells, potentially exerting a direct influence on the activation and functional state of immune cells by regulating oxidative stress (81). Jing Yan et al. experimentally demonstrated that GSR defects significantly impair neutrophil phagocytic function and reduce the migratory capacity of macrophages and eosinophils to the peritoneal cavity (82). However, the underlying mechanism linking the GSR gene to the immune microenvironment in BC remains unclear at present. We obtained some key genes from transcriptome sequencing. Since these genes only exist in the transcriptome data, we aim to explore whether they exhibit similar differential expression across cells and cell subpopulations using additional methods. Therefore, single-cell RNA sequencing (scRNA-seq) is required.

The application of machine learning in scRNA-seq has further deepened our understanding of gene expression dynamics in BC. By analyzing the gene expression patterns of distinct cell types identified, we can accurately pinpoint the specific cellular environments where these genes play a pivotal role (83). CD8+ T cells enhance antitumor immunity through antigen presentation and cell-mediated interferon-γ responses (84), thereby limiting tumor spread and improving prognosis (85, 86). Our findings demonstrate that ACADS expression is significantly upregulated in T cell subsets, suggesting it may perform important biological functions within these cellular subpopulations. Qi Wu et al. found that ACADS primarily influences antitumor immune responses by regulating the infiltration levels of T cell subsets within the tumor microenvironment (87), consistent with our findings.

Finally, we conducted drug prediction and molecular docking experiments on the potential disease biomarkers (ACADS, AIFM2, GSR, and PMAIP1). Through these experiments, we screened out disulfiram and eugenol as potential therapeutic drugs for BC and predicted their binding energy with the key genes ACADS, AIFM2, GSR, and PMAIP1. Disulfiram is a traditional medication used to treat alcohol dependence. Its anti-tumor mechanism in BC therapy involves processes such as apoptosis induction, reactive oxygen species generation, and metal ion regulation (88, 89). Eugenol is a naturally occurring phenolic compound that exhibits anti-proliferative effects against BC cells through multiple mechanisms, including the induction of apoptosis (90, 91), inhibition of cell proliferation (92, 93), and targeting of the E2F1/survivin pathway (94). Molecular docking results indicate that the predicted drug exhibits favorable binding energy with the key target, capable of forming a stable conformation. These findings may provide significant reference for personalized treatment of BC, with the potential to advance therapeutic strategies for BC. Molecular dynamics simulations serve as a critical computational biology tool. By simulating the temporal evolution of biomolecules at the atomic level, they validate the binding stability of protein-ligand complexes, analyze interactions (such as hydrogen bonds), and assess conformational changes. This complements static molecular docking results, enhancing the reliability of binding energy predictions (95). We selected the protein-ligand pairs with the optimal molecular docking binding energy (ACADS-Eugenol and AIFM2-Eugenol) for molecular dynamics simulations. Results indicate stable complex formation with robust hydrogen bonding interactions. Consequently, the small molecule Eugenol exhibits favorable binding to both AIFM2 and ACADS target proteins.

This study still has certain limitations. Firstly, regarding current public data, there is some heterogeneity in the data sources. For example, although all tumor samples were derived from BC tissues, considering the large number of BC subtypes, our transcriptome analysis data cover a relatively comprehensive range of these subtypes, including inflammatory BC and TNBC. Although batch effect correction was performed, batch effects or errors associated with current technologies and algorithms may still exist. Additionally, we conducted preliminary verification of the expression and function of key genes through cell experiments and observed a corresponding trend; however, the research conclusions and their generalization still require further verification. In the future, we plan to conduct multi-dimensional validation using additional human tumor samples and animal models to comprehensively assess the diagnostic value and molecular mechanisms of these BC biomarkers.

## Data Availability

The raw data supporting the conclusions of this article will be made available by the authors, without undue reservation.

## Supplementary material

Supplementary Figure 1 Validating model performance on the TCGA-BRCA RNA-seq dataset. **(A)** The efficacy of the model biomarkers was evaluated in the TCGA-BRCA RNA-seq dataset using a confusion matrix. **(B)** Verify the AUC value of the hub gene in the TCGA-BRCA RNA-seq dataset.

Supplementary Figure 2 Results of GSEA enrichment analysis for hub genes. Results of GSEA Enrichment Analysis for Hub Genes. **(A)** ACADS **(B)** AIFM2 **(C)** ARMCX1 **(D)** CKMT2 **(E)** ETFDH **(F)** GSR **(G)** HIBADH **(H)** IMMP2L **(I)** KMO **(J)** NIPSNAP3B **(K)** OXCT1 **(L)** PMAIP1 **(M)** VPS13D **(N)** PPIF **(O)** SDSL **(P)** SFXN1 **(Q)** PNKD **(R)** SFXN2

Supplementary Figure 3 Immunohistochemistry results of key hub genes from the HPA database. The protein expression levels of ACADS, ARMCX1, ETFDH, HIBADH, IMMP2L, NIPSNAP3B, and OXCT1 were significantly downregulated in BC tissue, whereas GSR, SDSL, SFXN1, and SFXN2 were upregulated.

## References

[1] Harbeck N Penault-Llorca F Cortes J Gnant M Houssami N Poortmans P . Breast cancer. Nat Rev Dis Primers. (2019) 5:66. doi: 10.1038/s41572-019-0111-2, PMID: 31548545

[2] Hong R Xu B . Breast cancer: an up-to-date review and future perspectives. Cancer Commun (London England). (2022) 42:913–36. doi: 10.1002/cac2.12358, PMID: 36074908 PMC9558690

[3] Zheng R Zhang S Zeng H Wang S Sun K Chen R . Cancer incidence and mortality in China, 2016. J Natl Cancer Center. (2022) 2:1–09. doi: 10.1016/j.jncc.2022.02.002, PMID: 39035212 PMC11256658

[4] Xiong N Wu H Yu Z . Advancements and challenges in triple-negative breast cancer: a comprehensive review of therapeutic and diagnostic strategies. Front Oncol. (2024) 14:1405491. doi: 10.3389/fonc.2024.1405491, PMID: 38863622 PMC11165151

[5] Turashvili G Brogi E . Tumor heterogeneity in breast cancer. Front Med (Lausanne). (2017) 4:227. doi: 10.3389/fmed.2017.00227, PMID: 29276709 PMC5727049

[6] Monzel AS Enríquez JA Picard M . Multifaceted mitochondria: moving mitochondrial science beyond function and dysfunction. Nat Metab. (2023) 5:546–62. doi: 10.1038/s42255-023-00783-1, PMID: 37100996 PMC10427836

[7] Hu Y Liu W Fang W Dong Y Zhang H Luo Q . Tumor energy metabolism: implications for therapeutic targets. Mol BioMed. (2024) 5:63. doi: 10.1186/s43556-024-00229-4, PMID: 39609317 PMC11604893

[8] Wang Q Yin X Huang X Zhang L Lu H . Impact of mitochondrial dysfunction on the antitumor effects of immune cells. Front Immunol. (2024) 15:1428596. doi: 10.3389/fimmu.2024.1428596, PMID: 39464876 PMC11502362

[9] Wang Q Du X Zhou B Li J Lu W Chen Q . Mitochondrial dysfunction is responsible for fatty acid synthase inhibition-induced apoptosis in breast cancer cells by PdpaMn. Biomedicine pharmacotherapy = Biomedecine pharmacotherapie. (2017) 96:396–403. doi: 10.1016/j.biopha.2017.10.008, PMID: 29031197

[10] Balliet RM Capparelli C Guido C Pestell TG Martinez-Outschoorn UE Lin Z . Mitochondrial oxidative stress in cancer-associated fibroblasts drives lactate production, promoting breast cancer tumor growth: understanding the aging and cancer connection. Cell Cycle (Georgetown Tex.). (2011) 10:4065–73. doi: 10.4161/cc.10.23.18254, PMID: 22129993 PMC3272288

[11] Zhen Y Yuan Z Zhang J Chen Y Fu Y Liu Y . Flubendazole induces mitochondrial dysfunction and DRP1-mediated mitophagy by targeting EVA1A in breast cancer. Cell Death Dis. (2022) 13:375. doi: 10.1038/s41419-022-04823-8, PMID: 35440104 PMC9019038

[12] Cook CC Kim A Terao S Gotoh A Higuchi M . Consumption of oxygen: a mitochondrial-generated progression signal of advanced cancer. Cell Death Dis. (2012) 3:e258. doi: 10.1038/cddis.2011.141, PMID: 22258408 PMC3270275

[13] Chen B Sun X Huang H Feng C Chen W Wu D . An integrated machine learning framework for developing and validating a diagnostic model of major depressive disorder based on interstitial cystitis-related genes. J Affect Disord. (2024) 359:22–32. doi: 10.1016/j.jad.2024.05.061, PMID: 38754597

[14] Jo Y Yeo M Dao T Kwon J Yi H Ryu D . Machine learning-featured Secretogranin V is a circulating diagnostic biomarker for pancreatic adenocarcinomas associated with adipopenia. Front Oncol. (2022) 12:942774. doi: 10.3389/fonc.2022.942774, PMID: 36059698 PMC9428794

[15] Vaziri-Moghadam A Foroughmand-Araabi M . Integrating machine learning and bioinformatics approaches for identifying novel diagnostic gene biomarkers in colorectal cancer. Sci Rep. (2024) 14:24786. doi: 10.1038/s41598-024-75438-6, PMID: 39433800 PMC11494190

[16] Yang M Yang H Ji L Hu X Tian G Wang B . A multi-omics machine learning framework in predicting the survival of colorectal cancer patients. Comput Biol Med. (2022) 146:105516. doi: 10.1016/j.compbiomed.2022.105516, PMID: 35468406

[17] Xiang J Liu S Chang Z Li J Liu Y Wang H . Integrating transcriptomics and machine learning for immunotherapy assessment in colorectal cancer. Cell Death Discov. (2024) 10:162. doi: 10.1038/s41420-024-01934-3, PMID: 38565865 PMC10987483

[18] Li X Lin Z Wang P Zhou C Xu J Lin J . Tetramethylpyrazine-Rhein Derivative inhibits the migration of canine inflammatory mammary carcinoma cells by mitochondrial damage-mediated apoptosis and cadherins downregulation. Biomedicine pharmacotherapy = Biomedecine pharmacotherapie. (2023) 162:114731. doi: 10.1016/j.biopha.2023.114731, PMID: 37086510

[19] Cruz-Gregorio A Aranda-Rivera AK Aparicio-Trejo OE Medina-Campos ON Sciutto E Fragoso G . α-Mangostin induces oxidative damage, mitochondrial dysfunction, and apoptosis in a triple-negative breast cancer model. Phytotherapy research: PTR. (2023) 37:3394–407. doi: 10.1002/ptr.7812, PMID: 37012651

[20] Xu M Zhou H Hu P Pan Y Wang S Liu L . Identification and validation of immune and oxidative stress-related diagnostic markers for diabetic nephropathy by WGCNA and machine learning. Front Immunol. (2023) 14:1084531. doi: 10.3389/fimmu.2023.1084531, PMID: 36911691 PMC9992203

[21] Xun D Li X Huang L Zhao Y Chen J Qi X . Machine learning-based analysis identifies a 13-gene prognostic signature to improve the clinical outcomes of colorectal cancer. J Gastrointest Oncol. (2024) 15:2100–16. doi: 10.21037/jgo-24-325, PMID: 39554586 PMC11565104

[22] Gao F Teng F Wan Y Zhang Q Huang J . The role of mitochondria-related genes in hepatocellular carcinoma prognosis: construction of prognostic models based on machine learning. Discover Oncol. (2025) 16:1407. doi: 10.1007/s12672-025-03216-5, PMID: 40715829 PMC12297210

[23] Becker K Savvides SN Keese M Schirmer RH Karplus PA . Enzyme inactivation through sulfhydryl oxidation by physiologic NO-carriers. Nature Structural Biology. (1998) 5:267–71. doi: 10.1038/nsb0498-267., PMID: 9546215

[24] Guan R Xiao R Zhao L Acton TB Gelinas C Montelione GT . Crystal Structure of human BFL-1 in complex with NOXA BH3 peptide, Northeast Structural Genomics Consortium Target HR2930. Worldwide Protein Data Bank. (2010) doi: 10.2210/pdb3mqp/pdb

[25] Wang Y Xu F Nichols CB Shi Y Hellinga HW Alspaugh JA . Structure-Guided Discovery of Potent Antifungals that Prevent Ras Signaling by Inhibiting Protein Farnesyltransferase. J Med Chem. (2022) 65:13753–70. doi: 10.1021/acs.jmedchem.2c00902., PMID: 36218371 PMC10755971

[26] Lan H Gao Y Hong T Chang Z Zhao Z Wang Y . Data from: Structural insight into 6-OH-FAD-dependent activation of hFSP1 for ferroptosis suppression. Cell Discovery. (2024) 10:88. doi: 10.1038/s41421-024-00723-7., PMID: 39160155 PMC11333494

[27] Filippi S Paccosi E Balzerano A Ferretti M Poli G Taborri J . CSA antisense targeting enhances anticancer drug sensitivity in breast cancer cells, including the triple-negative subtype. Cancers (Basel). (2022) 14:1687. doi: 10.3390/cancers14071687, PMID: 35406459 PMC8997023

[28] García-Ledo L Nuevo-Tapioles C Cuevas-Martín C Martínez-Reyes I Soldevilla B González-Llorente L . Overexpression of the ATPase inhibitory factor 1 favors a non-metastatic phenotype in breast cancer. Front Oncol. (2017) 7:69. doi: 10.3389/fonc.2017.00069, PMID: 28443245 PMC5385467

[29] Lucantoni F Salvucci M Dussmann H Prehn JHM . BCL(X)L and BCL2 increase mitochondrial dynamics in breast cancer cell: Evidence from functional and genetic studies. Biochim Biophys Acta Mol Cell Res. (2021) 1868:119095. doi: 10.1016/j.bbamcr.2021.119095, PMID: 34214511

[30] Cruz-Gregorio A Aranda-Rivera AK Aparicio-Trejo OE Medina-Campos ON Sciutto E Fragoso G . GK-1 induces oxidative stress, mitochondrial dysfunction, decreased membrane potential, and impaired autophagy flux in a mouse model of breast cancer. Antioxidants (Basel Switzerland). (2022) 12:56. doi: 10.3390/antiox12010056, PMID: 36670920 PMC9854788

[31] Wang S Chang Y Tzeng Y Wu C Wang Y Tseng L . Mitochondrial stress adaptation promotes resistance to aromatase inhibitor in human breast cancer cells via ROS/calcium up-regulated amphiregulin-estrogen receptor loop signaling. Cancer Lett. (2021) 523:82–99. doi: 10.1016/j.canlet.2021.09.043, PMID: 34610415

[32] Whitaker-Menezes D Martinez-Outschoorn UE Lin Z Ertel A Flomenberg N Witkiewicz AK . Evidence for a stromal-epithelial “lactate shuttle” in human tumors: MCT4 is a marker of oxidative stress in cancer-associated fibroblasts. Cell Cycle (Georgetown Tex.). (2011) 10:1772–83. doi: 10.4161/cc.10.11.15659, PMID: 21558814 PMC3142461

[33] Miller KD O’Connor S Pniewski KA Kannan T Acosta R Mirji G . Acetate acts as a metabolic immunomodulator by bolstering T-cell effector function and potentiating antitumor immunity in breast cancer. Nat Cancer. (2023) 4:1491–507. doi: 10.1038/s43018-023-00636-6, PMID: 37723305 PMC10615731

[34] Yang P Tsai E Hou M Lee Y Wang T . Global untargeted and individual targeted plasma metabolomics of breast cancer recurrence modified by hormone receptors. Breast Cancer (Tokyo Japan). (2024) 31:659–70. doi: 10.1007/s12282-024-01579-1, PMID: 38652345

[35] Bordonaro M . Further analysis of p300 in mediating effects of Butyrate in Colorectal Cancer Cells. J Cancer. (2020) 11:5861–66. doi: 10.7150/jca.47160, PMID: 32922528 PMC7477411

[36] Gálvez-Navas JM Molina-Montes E Rodríguez-Barranco M Ramírez-Tortosa M Gil Á Sánchez M . Molecular mechanisms linking genes and vitamins of the complex B related to one-carbon metabolism in breast cancer: an in silico functional database study. Int J Mol Sci. (2024) 25:8175. doi: 10.3390/ijms25158175, PMID: 39125744 PMC11311893

[37] Lin Y Lin L Fu F Wang C Hu A Xie J . Quantitative proteomics reveals stage-specific protein regulation of triple negative breast cancer. Breast Cancer Res Treat. (2021) 185:39–52. doi: 10.1007/s10549-020-05916-8, PMID: 32920739

[38] Chen D Feng X Lv Z Xu X Lu Y Wu W . ACADS acts as a potential methylation biomarker associated with the proliferation and metastasis of hepatocellular carcinomas. Aging. (2019) 11:8825–44. doi: 10.18632/aging.102292, PMID: 31652420 PMC6834414

[39] Schroth W Mürdter TE Schwab M . Unraveling the impact of drug metabolism on tamoxifen response in breast cancer. Cancer epidemiology Biomarkers prevention: Publ Am Assoc Cancer Research cosponsored by Am Soc Prev Oncol. (2025) 34:221–23. doi: 10.1158/1055-9965.EPI-24-1617, PMID: 39910985

[40] Wu X Hawse JR Subramaniam M Goetz MP Ingle JN Spelsberg TC . The tamoxifen metabolite, endoxifen, is a potent antiestrogen that targets estrogen receptor alpha for degradation in breast cancer cells. Cancer Res. (2009) 69:1722–27. doi: 10.1158/0008-5472.CAN-08-3933, PMID: 19244106

[41] Murray GI Patimalla S Stewart KN Miller ID Heys SD . Profiling the expression of cytochrome P450 in breast cancer. Histopathology. (2010) 57:202–11. doi: 10.1111/j.1365-2559.2010.03606.x, PMID: 20716162

[42] Sneha S Baker SC Green A Storr S Aiyappa R Martin S . Intratumoural cytochrome P450 expression in breast cancer: impact on standard of care treatment and new efforts to develop tumour-selective therapies. Biomedicines. (2021) 9:290. doi: 10.3390/biomedicines9030290, PMID: 33809117 PMC7998590

[43] Zhao B Xin Z Ren P Wu H . The role of PPARs in breast cancer. Cells. (2022) 12:130. doi: 10.3390/cells12010130, PMID: 36611922 PMC9818187

[44] Augimeri G Giordano C Gelsomino L Plastina P Barone I Catalano S . The role of PPARγ Ligands in breast cancer: from basic research to clinical studies. Cancers (Basel). (2020) 12:2623. doi: 10.3390/cancers12092623, PMID: 32937951 PMC7564201

[45] Karande AA Sridhar L Gopinath KS Adiga PR Riboflavin carrier protein: a serum and tissue marker for breast carcinoma. Int J Cancer. (2001) 95:277–81. doi: 10.1002/1097-0215(20010920)95:5, PMID: 11494224

[46] Bartmann L Schumacher D von Stillfried S Sternkopf M Alampour-Rajabi S van Zandvoort MAMJ . Evaluation of riboflavin transporters as targets for drug delivery and theranostics. Front Pharmacol. (2019) 10:79. doi: 10.3389/fphar.2019.00079, PMID: 30787877 PMC6372557

[47] Schömel N Hancock SE Gruber L Olzomer EM Byrne FL Shah D . UGCG influences glutamine metabolism of breast cancer cells. Sci Rep. (2019) 9:15665. doi: 10.1038/s41598-019-52169-7, PMID: 31666638 PMC6821892

[48] Albogami SM Asiri Y Asiri A Alnefaie AA Alnefaie S . Effects of neoadjuvant therapies on genetic regulation of targeted pathways in ER+ primary ductal breast carcinoma: A meta-analysis of microarray datasets. Saudi Pharm journal: SPJ: Off Publ Saudi Pharm Soc. (2021) 29:656–69. doi: 10.1016/j.jsps.2021.04.027, PMID: 34400859 PMC8347676

[49] Luo J Cao D Hu C Liang Z Zhang Y Lai J . Lymphatic metastasis-associated circRNA–miRNA–mRNA network for exploring the pathogenesis and therapeutic target of triple negative breast cancer based on whole-transcriptome sequencing analysis: an experimental verification study. J Transl Med. (2022) 20:508. doi: 10.1186/s12967-022-03728-6, PMID: 36335337 PMC9636725

[50] Cai L Shao X Mao X Fu Y Yang Q . Triple-helix β-glucan-based self-assemblies, synthesis, characterization and anticarcinogenic effect. Int J Biol Macromol. (2025) 286:138427. doi: 10.1016/j.ijbiomac.2024.138427, PMID: 39653201

[51] Sukocheva OA . Expansion of sphingosine kinase and sphingosine-1-phosphate receptor function in normal and cancer cells: from membrane restructuring to mediation of estrogen signaling and stem cell programming. Int J Mol Sci. (2018) 19:420. doi: 10.3390/ijms19020420, PMID: 29385066 PMC5855642

[52] Huang C Su L Chen Y Wu S Sun R Xu Q . Ceramide kinase confers tamoxifen resistance in estrogen receptor-positive breast cancer by altering sphingolipid metabolism. Pharmacol Res. (2023) 187:106558. doi: 10.1016/j.phrs.2022.106558, PMID: 36410675

[53] Corsetto PA Zava S Rizzo AM Colombo I . The critical impact of sphingolipid metabolism in breast cancer progression and drug response. Int J Mol Sci. (2023) 24:2107. doi: 10.3390/ijms24032107, PMID: 36768427 PMC9916652

[54] Pedersen L Panahandeh P Siraji MI Knappskog S Lønning PE Gordillo R . Golgi-localized PAQR4 mediates antiapoptotic ceramidase activity in breast cancer. Cancer Res. (2020) 80:2163–74. doi: 10.1158/0008-5472.CAN-19-3177, PMID: 32291319

[55] Bataller M Sánchez-García A Garcia-Mayea Y Mir C Rodriguez I LLeonart ME . The role of sphingolipids metabolism in cancer drug resistance. Front Oncol. (2021) 11:807636. doi: 10.3389/fonc.2021.807636, PMID: 35004331 PMC8733468

[56] Gong C Jiao C Liang H Ma Y Wu Q Xie Y . Exome-based amino acid optimization: A dietary strategy to satisfy human nutritional demands and enhance muscle strength in breast tumor mice undergoing chemotherapy. J Agric Food Chem. (2024) 72:7089–99. doi: 10.1021/acs.jafc.3c07256, PMID: 38512774

[57] Zhou C He X Tong C Li H Xie C Wu Y . Cancer-associated adipocytes promote the invasion and metastasis in breast cancer through LIF/CXCLs positive feedback loop. Int J Biol Sci. (2022) 18:1363–80. doi: 10.7150/ijbs.65227, PMID: 35280694 PMC8898379

[58] Mosly D MacLeod K Moir N Turnbull A Sims AH Langdon SP . Variation in IL6ST cytokine family function and the potential of IL6 trans-signalling in ERα positive breast cancer cells. Cell Signal. (2023) 103:110563. doi: 10.1016/j.cellsig.2022.110563, PMID: 36565897

[59] Niu XL Wang Y Yao Z Duan H Li Z Liu W . Autocrine interferon-γ may affect Malignant behavior and sensitivity to tamoxifen of MCF-7 via estrogen receptor β subtype. Oncol Rep. (2015) 34:3120–30. doi: 10.3892/or.2015.4294, PMID: 26397740

[60] Bazhabayi M Qiu X Li X Yang A Wen W Zhang X . CircGFRA1 facilitates the Malignant progression of HER-2-positive breast cancer via acting as a sponge of miR-1228 and enhancing AIFM2 expression. J Cell Mol Med. (2021) 25:10248–56. doi: 10.1111/jcmm.16963, PMID: 34668628 PMC8572792

[61] He J McLaughlin RP van der Beek L Canisius S Wessels L Smid M . Integrative analysis of genomic amplification-dependent expression and loss-of-function screen identifies ASAP1 as a driver gene in triple-negative breast cancer progression. Oncogene. (2020) 39:4118–31. doi: 10.1038/s41388-020-1279-3, PMID: 32235890 PMC7220851

[62] Wang P Yu W Hu Z Jia L Iyer VR Sanders BG . Involvement of JNK/p73/NOXA in vitamin E analog-induced apoptosis of human breast cancer cells. Mol Carcinog. (2008) 47:436–45. doi: 10.1002/mc.20400, PMID: 18058804

[63] Rajaram S Synnott NC Crown J Madden SF Duffy MJ . Targeting mutant p53 with arsenic trioxide: A preclinical study focusing on triple negative breast cancer. Transl Oncol. (2024) 46:102025. doi: 10.1016/j.tranon.2024.102025, PMID: 38870678 PMC11225897

[64] Raza U Saatci Ö Uhlmann S Ansari SA Eyüpoğlu E Yurdusev E . The miR-644a/CTBP1/p53 axis suppresses drug resistance by simultaneous inhibition of cell survival and epithelial-mesenchymal transition in breast cancer. Oncotarget. (2016) 7:49859–77. doi: 10.18632/oncotarget.10489, PMID: 27409664 PMC5226553

[65] Zhang Y Xu H Han X Yu Q Zheng L Xiao H . PMAIP1-mediated glucose metabolism and its impact on the tumor microenvironment in breast cancer: Integration of multi-omics analysis and experimental validation. Transl Oncol. (2025) 52:102267. doi: 10.1016/j.tranon.2024.102267, PMID: 39740516 PMC11750568

[66] Lee J Song J Yoo W Choi H Jung D Choi E . Therapeutic potential of anti-ErbB3 chimeric antigen receptor natural killer cells against breast cancer. Cancer immunology immunotherapy: CII. (2025) 74:73. doi: 10.1007/s00262-024-03923-y, PMID: 39751931 PMC11698710

[67] Li F Gao C Huang Y Qiao Y Xu H Liu S . Unraveling the breast cancer tumor microenvironment: crucial factors influencing natural killer cell function and therapeutic strategies. Int J Biol Sci. (2025) 21:2606–28. doi: 10.7150/ijbs.108803, PMID: 40303301 PMC12035885

[68] Hagerling C Gonzalez H Salari K Wang C Lin C Robles I . Immune effector monocyte-neutrophil cooperation induced by the primary tumor prevents metastatic progression of breast cancer. Proc Natl Acad Sci U S. (2019) 116:21704–14. doi: 10.1073/pnas.1907660116, PMID: 31591235 PMC6815161

[69] McDowell SAC Milette S Doré S Yu MW Sorin M Wilson L . Obesity alters monocyte developmental trajectories to enhance metastasis. J Exp Med. (2023) 220:e20220509. doi: 10.1084/jem.20220509, PMID: 37166450 PMC10182775

[70] Wei C Tsai I Wu C Hung W Hsuan C Yu T . Elevated plasma level of neutrophil gelatinase-associated lipocalin (NGAL) in patients with breast cancer. Int J Med Sci. (2021) 18:2689–96. doi: 10.7150/ijms.58789, PMID: 34104101 PMC8176172

[71] Kim HS Kim MG Min K Jung US Kim D . High MMP-11 expression associated with low CD8+ T cells decreases the survival rate in patients with breast cancer. PloS One. (2021) 16:e252052. doi: 10.1371/journal.pone.0252052, PMID: 34038440 PMC8153507

[72] Wang C Zhang L Ren L Zhang G Wan A Xiong S . A novel pyroptosis-related indicator of immune infiltration features and prognosis in breast cancer. Front Oncol. (2022) 12:961500. doi: 10.3389/fonc.2022.961500, PMID: 36158689 PMC9491236

[73] Kresovich JK O’Brien KM Xu Z Weinberg CR Sandler DP Taylor JA . Circulating leukocyte subsets before and after a breast cancer diagnosis and therapy. JAMA Netw Open. (2024) 7:e2356113. doi: 10.1001/jamanetworkopen.2023.56113, PMID: 38358741 PMC10870180

[74] Chakraborty D Ivan C Amero P Khan M Rodriguez-Aguayo C Başağaoğlu H . Explainable artificial intelligence reveals novel insight into tumor microenvironment conditions linked with better prognosis in patients with breast cancer. Cancers (Basel). (2021) 13:3450. doi: 10.3390/cancers13143450, PMID: 34298668 PMC8303703

[75] Gadde M Mehrabi-Dehdezi M Debeb BG Woodward WA Rylander MN . Influence of macrophages on vascular invasion of inflammatory breast cancer emboli measured using an *in vitro* microfluidic multi-cellular platform. Cancers (Basel). (2023) 15:4883. doi: 10.3390/cancers15194883, PMID: 37835577 PMC10571588

[76] Luo Z Huang X Xu X Wei K Zheng Y Gong K . Decreased LDHB expression in breast tumor cells causes NK cell activation and promotes tumor progression. Cancer Biol Med. (2024) 21:513–40. doi: 10.20892/j.issn.2095-3941.2023.0382, PMID: 38525901 PMC11208901

[77] Chan IS Knútsdóttir H Ramakrishnan G Padmanaban V Warrier M Ramirez JC . Cancer cells educate natural killer cells to a metastasis-promoting cell state. J Cell Biol. (2020) 219:e202001134. doi: 10.1083/jcb.202001134, PMID: 32645139 PMC7480097

[78] Hamilton E Clay TM Blackwell KL . New perspectives on zoledronic acid in breast cancer: potential augmentation of anticancer immune response. Cancer Invest. (2011) 29:533–41. doi: 10.3109/07357907.2011.605413, PMID: 21843051

[79] Boufea K González-Huici V Lindberg M Symeonides S Oikonomidou O Batada NN . Single-cell RNA sequencing of human breast tumour-infiltrating immune cells reveals a γδ T-cell subtype associated with good clinical outcome. Life Sci Alliance. (2021) 4:e202000680. doi: 10.26508/lsa.202000680, PMID: 33268347 PMC7723295

[80] Schmidt H Ge Y Hartmann FJ Conrad H Klug F Nittel S . HLA Class II tetramers reveal tissue-specific regulatory T cells that suppress T-cell responses in breast carcinoma patients. Oncoimmunology. (2013) 2:e24962. doi: 10.4161/onci.24962, PMID: 23894725 PMC3716760

[81] Jiang H Ren Y Yu J Hu S Zhang J . Analysis of lactate metabolism-related genes and their association with immune infiltration in septic shock via bioinformatics method. Front Genet. (2023) 14:1223243. doi: 10.3389/fgene.2023.1223243, PMID: 37564869 PMC10410269

[82] Yan J Ralston MM Meng X Bongiovanni KD Jones AL Benndorf R . Glutathione reductase is essential for host defense against bacterial infection. Free Radical Biol Med. (2013) 61:320–32. doi: 10.1016/j.freeradbiomed.2013.04.015, PMID: 23623936 PMC3749296

[83] He Y You G Zhou Y Ai L Liu W Meng X . Integrative machine learning of glioma and coronary artery disease reveals key tumour immunological links. J Cell Mol Med. (2025) 29:e70377. doi: 10.1111/jcmm.70377, PMID: 39868675 PMC11770474

[84] Jiang J Zhao Y . CD8(+) T lymphocyte coexpression genes correlate with immune microenvironment and overall survival in breast cancer. J Oncol. (2021) 2021:5533923. doi: 10.1155/2021/5533923, PMID: 33854546 PMC8019641

[85] Ma H Shi L Zheng J Zeng L Chen Y Zhang S . Advanced machine learning unveils CD8 + T cell genetic markers enhancing prognosis and immunotherapy efficacy in breast cancer. BMC Cancer. (2024) 24:1222. doi: 10.1186/s12885-024-12952-w, PMID: 39354417 PMC11446097

[86] Liu F Lang R Zhao J Zhang X Pringle GA Fan Y . CD8^+^ cytotoxic T cell and FOXP3^+^ regulatory T cell infiltration in relation to breast cancer survival and molecular subtypes. Breast Cancer Res Treat. (2011) 130:645–55. doi: 10.1007/s10549-011-1647-3, PMID: 21717105

[87] Wu Q Yan T Chen Y Chang J Jiang Y Zhu D . Integrated analysis of expression and prognostic values of acyl-coA dehydrogenase short-chain in colorectal cancer. Int J Med Sci. (2021) 18:3631–43. doi: 10.7150/ijms.63953, PMID: 34790035 PMC8579304

[88] Fasehee H Dinarvand R Ghavamzadeh A Esfandyari-Manesh M Moradian H Faghihi S . Delivery of disulfiram into breast cancer cells using folate-receptor-targeted PLGA-PEG nanoparticles: *in vitro* and *in vivo* investigations. J Nanobiotechnology. (2016) 14:32. doi: 10.1186/s12951-016-0183-z, PMID: 27102110 PMC4839071

[89] Wiggins HL Wymant JM Solfa F Hiscox SE Taylor KM Westwell AD . Disulfiram-induced cytotoxicity and endo-lysosomal sequestration of zinc in breast cancer cells. Biochem Pharmacol. (2015) 93:332–42. doi: 10.1016/j.bcp.2014.12.014, PMID: 25557293 PMC4318799

[90] Al-Sharif I Remmal A Aboussekhra A . Eugenol triggers apoptosis in breast cancer cells through E2F1/survivin down-regulation. BMC Cancer. (2013) 13:600. doi: 10.1186/1471-2407-13-600, PMID: 24330704 PMC3931838

[91] Abdullah ML Al-Shabanah O Hassan ZK Hafez MM . Eugenol-induced autophagy and apoptosis in breast cancer cells via PI3K/AKT/FOXO3a pathway inhibition. Int J Mol Sci. (2021) 22:9243. doi: 10.3390/ijms22179243, PMID: 34502165 PMC8430664

[92] Abdullah ML Hafez MM Al-Hoshani A Al-Shabanah O . Anti-metastatic and anti-proliferative activity of eugenol against triple negative and HER2 positive breast cancer cells. BMC Complement Altern Med. (2018) 18:321. doi: 10.1186/s12906-018-2392-5, PMID: 30518369 PMC6282398

[93] Ma M Ma Y Zhang G Liao R Jiang X Yan X . Eugenol alleviated breast precancerous lesions through HER2/PI3K-AKT pathway-induced cell apoptosis and S-phase arrest. Oncotarget. (2017) 8:56296–310. doi: 10.18632/oncotarget.17626, PMID: 28915591 PMC5593562

[94] Al-Kharashi LA Bakheet T AlHarbi WA Al-Moghrabi N Aboussekhra A . Eugenol modulates genomic methylation and inactivates breast cancer-associated fibroblasts through E2F1-dependent downregulation of DNMT1/DNMT3A. Mol Carcinog. (2021) 60:784–95. doi: 10.1002/mc.23344, PMID: 34473867

[95] Grewal S Deswal G Grewal AS Guarve K . Molecular dynamics simulations: Insights into protein and protein ligand interactions. Adv Pharmacol (San Diego Calif.). (2025) 103:139–62. doi: 10.1016/bs.apha.2025.01.007, PMID: 40175039

