# Supplementary figures and images for: Development and validation of a machine learning-driven mitochondrial gene signature for the diagnosis of breast cancer
Source: Front Immunol. 2025 Dec 3;16:1712089. doi: 10.3389/fimmu.2025.1712089 (PMC12708286; doi:10.3389/fimmu.2025.1712089)

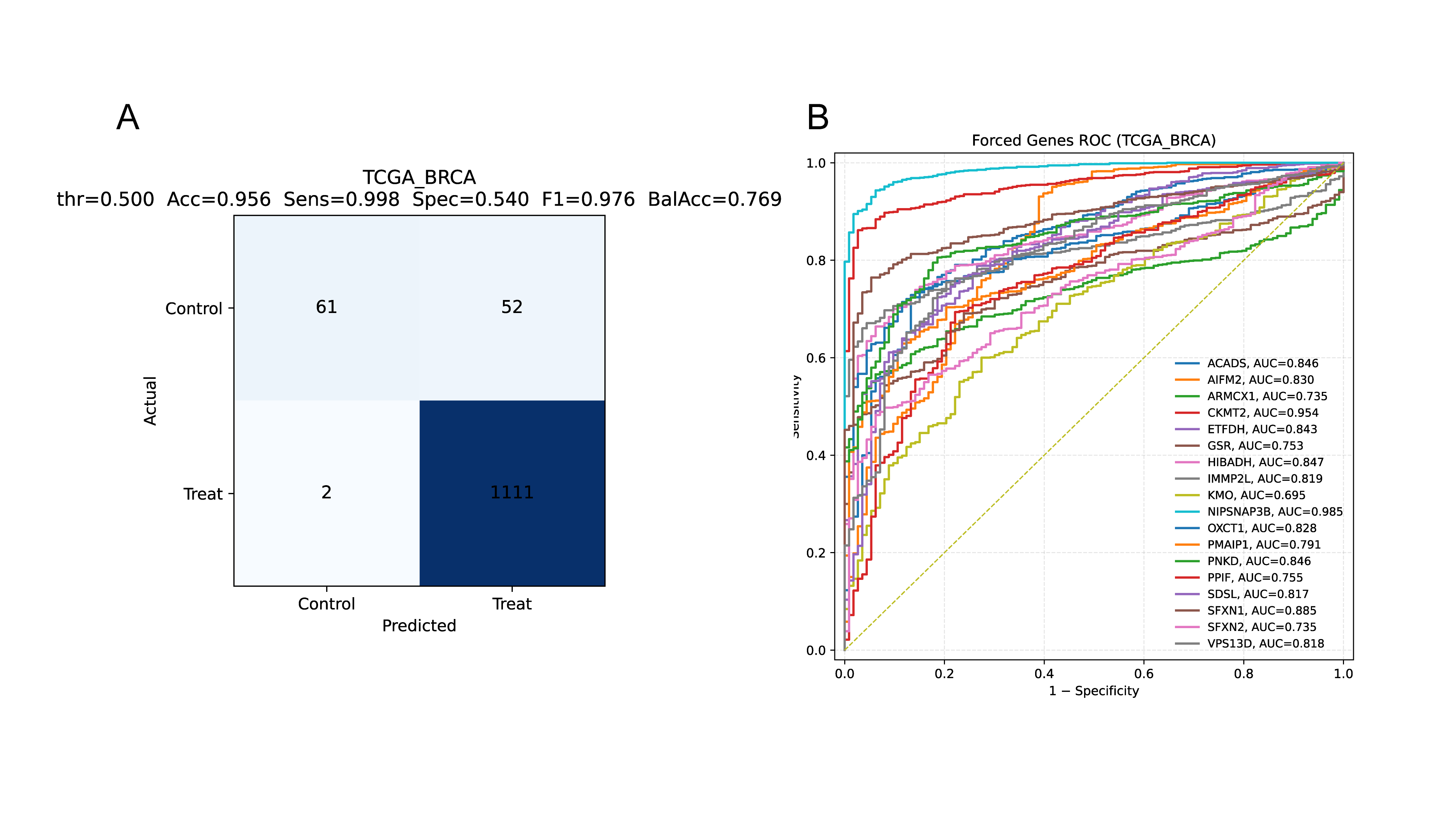

Supplement: Supplementary Figure 1 — Validating model performance on the TCGA-BRCA RNA-seq dataset. (A) The efficacy of the model biomarkers was evaluated in the TCGA-BRCA RNA-seq dataset using a confusion matrix. (B) Verify the AUC value of the hub gene in the TCGA-BRCA RNA-seq dataset. [file Image1.tif]

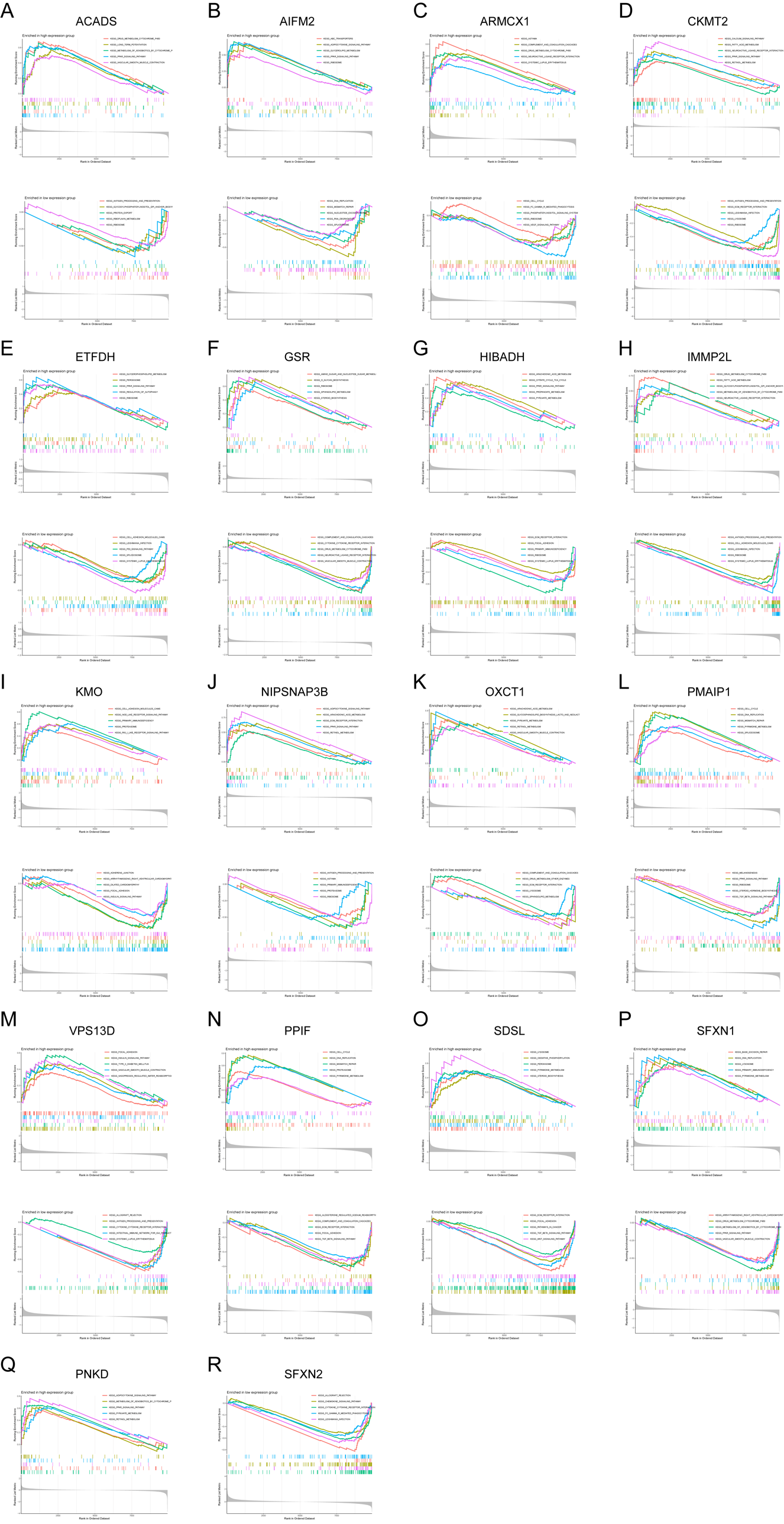

Supplement: Supplementary Figure 2 — Results of GSEA enrichment analysis for hub genes. Results of GSEA Enrichment Analysis for Hub Genes. (A) ACADS (B) AIFM2 (C) ARMCX1 (D) CKMT2 (E) ETFDH (F) GSR (G) HIBADH (H) IMMP2L (I) KMO (J) NIPSNAP3B (K) OXCT1 (L) PMAIP1 (M) VPS13D (N) PPIF (O) SDSL (P) SFXN1 (Q) PNKD (R) SFXN2 [file Image2.tif]

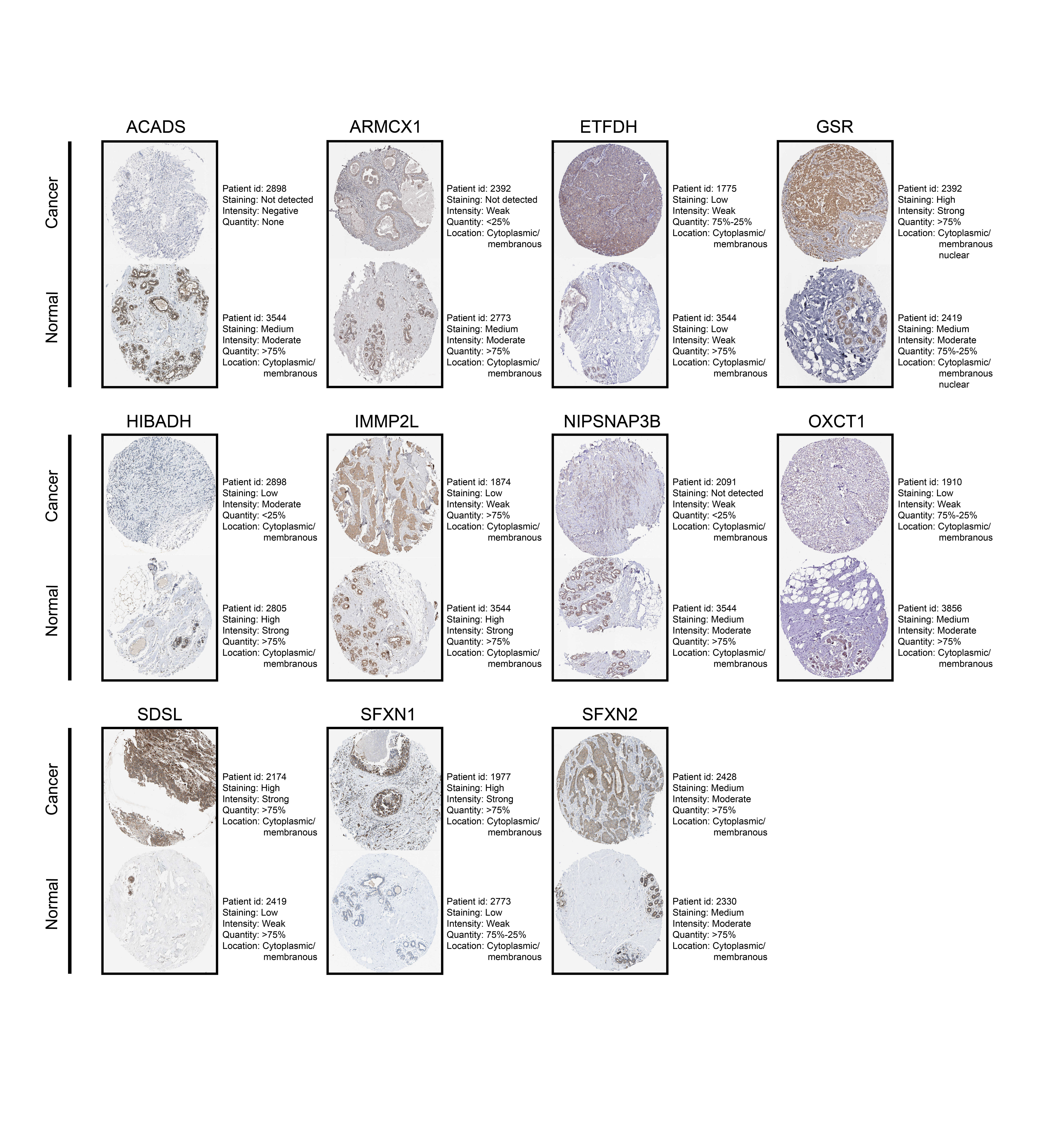

Supplement: Supplementary Figure 3 — Immunohistochemistry results of key hub genes from the HPA database. The protein expression levels of ACADS, ARMCX1, ETFDH, HIBADH, IMMP2L, NIPSNAP3B, and OXCT1 were significantly downregulated in BC tissue, whereas GSR, SDSL, SFXN1, and SFXN2 were upregulated. [file Image3.jpeg]
